# Mimicking The Photosynthetic Special Pair With Aluminum(III) Porphyrin Homodimer – Fullerene (C_60_) Supramolecular Assemblies Capable of Long‐Lived Charge Separation

**DOI:** 10.1002/chem.70853

**Published:** 2026-03-13

**Authors:** Stefan Charon, Jatan K. Sharma, Niloofar Zarrabi, Peyton Ellis, Paul A. Karr, Art van der Est, Francis D'Souza, Prashanth K. Poddutoori

**Affiliations:** ^1^ Department of Chemistry and Biochemistry University of Minnesota Duluth Duluth Minnesota USA; ^2^ Department of Chemistry University of North Texas Denton Texas USA; ^3^ Department of Physical Sciences and Mathematics Wayne State College Wayne Nebraska USA; ^4^ Department of Chemistry Brock University St. Catharines Ontario Canada

**Keywords:** aluminum(III) porphyrin, charge separation, fullerene C_60_, homodimer, special pair mimic

## Abstract

The axial‐bonding ability of aluminum(III) porphyrin has been employed to construct two covalently linked homodimer systems. The center‐to‐center distance between the two porphyrins, which are connected via an oxalic bridge is ∼6.90 Å. Unlike most other *meso‐*substituted porphyrins, which have aromatic substituents, the Al porphyrins were decorated with propyl or heptyl side groups to tune their electronic properties. Further, the Lewis acidity of the Al center was exploited to coordinate the Lewis base, imidazole, appended to the electron acceptor, C_60_, to construct a self‐assembled supramolecular homodimer‐C_60_ donor‐acceptor conjugate. Steady state optical studies show that the exciton coupling between porphyrins is weak, and time‐resolved electron paramagnetic resonance (TREPR) measurements in the glass phase indicate that the triplet state of the dimer remains localized. When C_60_ is coordinated to the monomers and dimers, femtosecond and nanosecond transient absorption studies and TREPR measurements show that electron transfer between the porphyrin and fullerene occurs. Despite the weak coupling between the two porphyrins of the dimer, the presence of a second porphyrin enhances both the yield and lifetime of the charge‐separated state. Remarkably, the resulting charge‐separated lifetimes found to be between 2–5 µs in the investigated reaction center mimics.

## Graphical abstract

The axial‐bonding of aluminum(III) porphyrins was used to create two covalently linked special‐pair mimics to probe charge‐transfer between a porphyrin dimer and a C_60_ unit for artificial photosynthesis. Although excitonic coupling between the porphyrins is weak, incorporation of a second porphyrin markedly increases both the yield and lifetime of the charge‐separated state.

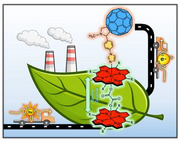

## 1 Introduction

The photosynthetic apparatus in plants, algae, and bacteria consists of multiple complex molecular components that operate together to convert solar energy into chemical energy [1, 2, 3, 4, 5, 6, 7, 8]. A major research objective has been to develop artificial systems that mimic the natural photosynthetic apparatus, to help meet future energy needs and mitigate environmental harm from fossil fuel combustion. In recent decades, researchers have developed various models of the natural photosynthetic apparatus for solar energy conversion and storage [9, 10, 11, 12, 13, 14, 15, 16, 17, 18, 19, 20, 21, 22, 23, 24, 25, 26, 27, 28]. Among these components, the chlorophyll special pair [29] is of particular interest because of its essential role in connecting the light‐harvesting antenna system to the reaction center complex and in facilitating long‐lived charge separation. The excitonic coupling in the special pair leads to a red shift of its absorbance so that it acts as a trap for the excitation energy. In the electron transfer chain, it acts as an electron donor, transferring an electron to either pheophytin or a chlorophyll acceptor. The delocalization of the charge over the two π‐systems of the resulting cation radical stabilizes the initial charge‐separated state. This stabilization slows charge recombination, providing additional time for the subsequent electron transfer steps, ultimately achieving a long‐lived charge‐separated state with high quantum yield.

Many impressive systems have been designed to mimic the special pair, primarily using porphyrin derivatives [9, 10, 30, 31, 32, 33, 34, 35, 36, 37]. Porphyrins are structurally similar to chlorophyll, and they possess versatile photophysical and redox properties [38, 39, 40, 41]. To mimic the cofacial arrangement of the special pair, two porphyrins are linked through covalently bound or coordinated bridging groups in such a way that their π‐π systems face each other. The bridging groups can be attached either on the periphery of the porphyrin ring through the β‐pyrrolic or *meso*‐positions or axially through the central element. Because the special pairs in photosynthesis are comprised of two identical or nearly identical chlorophylls, most of the model complexes are homodimers (that is, identical porphyrins). However, heterodimers (two different porphyrins) have also been reported [42, 43, 44, 45, 46]. These dimers have been studied primarily as model compounds for gaining insight into how the electronic and optical properties of coupled chromophores depend on distance and orientation. In contrast, relatively little attention has been paid to the use of these dimers as components of donor‐acceptor complexes to mimic the primary charge separation process of photosynthesis. Synthesizing such an analog of the special pair‐acceptor represents a significant step toward developing artificial photosynthetic devices for renewable solar energy production.

With the above objective in mind, we recently designed two heterodimers composed of an electron‐donor octaethylaluminum(III) porphyrin and an electron‐acceptor octaethylphosphorus(V) porphyrin, linked by μ‐oxo and phenyl bridges [47, 48, 49]. The μ‐oxo‐bridged heterodimer exhibited strong exciton coupling with charge transfer character. We attempted to promote subsequent electron transfer by attaching an electron donor to the Al center through Lewis acid‐base interactions, but these efforts were unsuccessful due to the weak Lewis acidity of the Al center. The eight ethyl groups on the porphyrin  β‐pyrrole positions increased the electron density and suppressed the Lewis acidity of the Al center, thus lowering its ability to form metal‐ligand axial coordination with Lewis base nitrogen ligands such as pyridine or imidazole. To enhance the Lewis acidity of the Al center, here, we have developed two new homodimers based on tetraalkylaluminum(III)porphyrin in which four alkyl groups are attached to the four meso‐positions rather than at the  β‐pyrrollic positions. The ability of the aluminum(III) porphyrins to form axial covalent bonds with carboxylic acids [50] was exploited to obtain homodimers. With the covalently bound homodimer in hand, the Lewis acidity of the Al center [51, 52, 53, 54, 55] was employed to coordinate imidazole appended C_60_ (C_60_‐Im) to the dimer. C_60_ was chosen due to its excellent electron‐accepting ability [56, 57, 58]. Also, it has been shown that porphyrin‐C_60_ donor‐acceptor complexes are an exceptional combination for building artificial photosynthetic systems [52, 59, 60, 61, 62, 63, 64, 65, 66]. We demonstrate that (i) the *meso*‐alkyl substitution does not compromise the Lewis acidity of the Al center, allowing C_60_‐Im to coordinate efficiently, and (ii) light‐induced electron transfer from the dimer to the coordinated C_60_‐Im unit occurs upon photoexcitation, leading to charge‐separated states. Time‐resolved electron paramagnetic resonance and femtosecond pump‐probe studies were performed to characterize the nature of the excited states, the exciton coupling between the two porphyrin entities, and the charge‐separation and charge‐recombination processes.

## 2 Results and Discussion

### 2.1 Synthesis and Characterization

**SCHEME 1:**
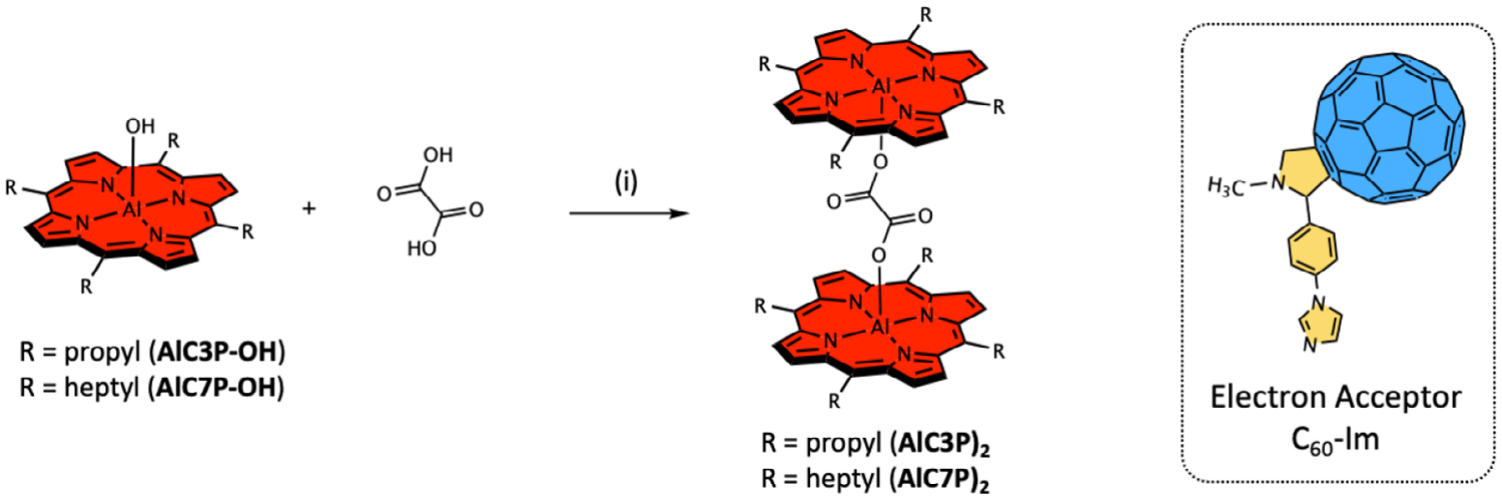
Synthesis of the studied homodimers. *Reaction conditions*: (i) Stirred in CH_2_Cl_2_ at room temperature under N_2_.

**SCHEME 2:**
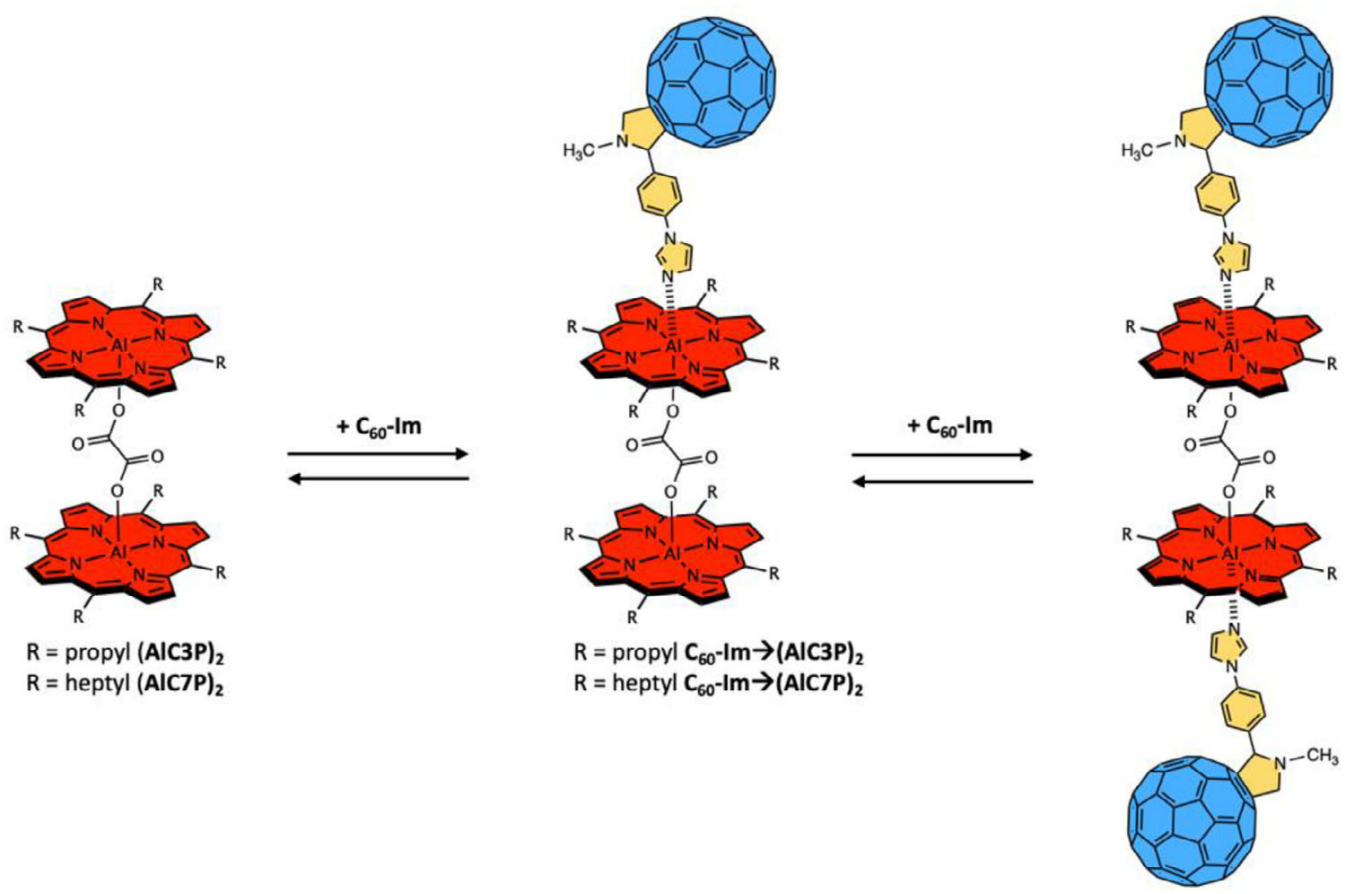
Self‐assembly of the supramolecular conjugates C_60_‐Im→(AlC3P)_2_ and C_60_‐Im→(AlC7P)_2_ in noncoordinating solvents.

The chemicals and solvents were procured from Alfa–Aesar, Acros Organics, Fisher Chemical, Sigma–Aldrich, and Tokyo Chemical Industry (TCI). Chromatographic materials were acquired from Sigma–Aldrich or SiliCycle. The complete synthesis details are given in the Supporting Information. The synthesis of precursor free‐base porphyrin H_2_C3P and its aluminum(III) derivative AlC3P‐OH was prepared using a modified reported procedure, see Scheme S1 [67]. The synthesis of precursor porphyrins, H_2_C7P and AlC7P‐OH, was reported elsewhere [68]. The reference compounds AlC3P and AlC7P are prepared according to Scheme S2. The dyads C_60_‐Im→AlC3P and C_60_‐Im→AlC7P were self‐assembled as per Scheme S3. Finally, the homodimers (AlC3P)_2_ and (AlC7P)_2_ and self‐assembled triads C_60_‐Im→(AlC3P)_2_ and C_60_‐Im→(AlC7P)_2_ were obtained according to Schemes 1 and 2. It is important to mention that the self‐assembly strategy shown in Scheme 2 also produces tetrads. However, this can be controlled by stoichiometric ratios of the homodimer and C_60_‐Im units, and under the investigated experimental conditions, we believe the triad system is the major complex. Therefore, the focus is on the triad systems, and the tetrads are outside the scope of this paper.

The initial structural characterization of the investigated compounds was carried out by ESI mass spectrometry, see Figures S1, S2. Each porphyrin showed an intense mass peak (*m*/*z*) corresponding to either the [M]^+^ or [M‐axial unit]^+^ ion. The IR spectra of AlC3P, AlC7P, (AlC3P)_2_, and (AlC7P)_2_ are shown in Figure S3. The two dimers (AlC3P)_2_, and (AlC7P)_2_ reveal peaks arising from both the porphyrin and bridging oxalate units. The presence of the carboxylate stretching around 1650 cm^−1^ [69] confirms formation of the dimers, that is, the two porphyrins are connected through an oxalate linker. The ^1^H NMR spectra are shown in Figures S4–S7, and the data are summarized in the Experimental Section, see Supporting Information. The free‐base porphyrins, H_2_C3P and H_2_C7P, manifested all the characteristic peaks such as the pyrrolic protons in the aromatic region [70], the aliphatic propyl or heptyl protons appear between 0.5–5.00 ppm, and lastly the inner NH protons appear at ∼−2.60 ppm. Upon Al insertion, the inner NH peak disappearance confirms the formation of aluminum(III) porphyrin. At this stage, the resulting porphyrin has two functionalities: (i) an active axial hydroxide group, and (ii) a Lewis acidic Al center. The hydroxide functional group was employed to construct the investigated homodimer molecules. In the later step, Lewis acid‐base interactions were used to build supramolecular homodimer‐C_60_ systems. The selection of alkyl units (propyl and heptyl) was to minimize the repulsions between *meso*‐substitutions as well as increase the stability of the dimer due to dispersion forces.

### 2.2 UV/Vis Absorption Studies

**FIGURE 1:**
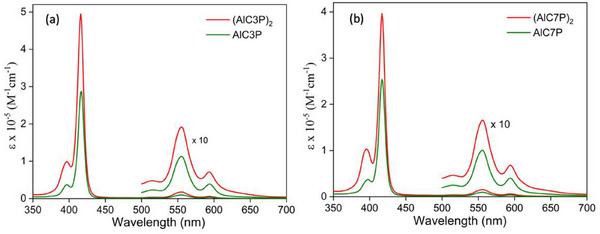
UV‐visible absorption spectra of (a) (AlC3P)_2_ and AlC3P (b) (AlC7P)_2_ and AlC7P in CH_2_Cl_2_.

UV−visible spectra of the homodimers (AlC3P)_2_ and (AlC7P)_2_ and the monomers AlC3P and AlC7P measured in CH_2_Cl_2_ are presented in Figure 1, and the peak positions (*λ*, nm) and their molar extinction coefficients (*ε*, M^−1^cm^−1^) are listed in Table 1. As shown in Figure 1a, b, the two monomers AlC3P and AlC7P (green spectra) show a sharp Soret band at 417 nm and two Q‐bands at 555 and 594 nm. The same bands are also observed for the two homodimers (AlC3P)_2_ and (AlC7P)_2,_ (red spectra) at essentially the same wavelengths. The *ε* values of the dimers are approximately twice as large as those of the monomers because there are two porphyrin units per molecule in the dimer, that is, the absorbance of the monomers and dimers is essentially the same when normalized to the concentration of porphyrin units. Overall, the similar spectral profiles of monomer and dimer indicate very weak or negligible exciton coupling [71] between two porphyrins π‐systems in the dimer molecules.

**TABLE 1:** UV‐visible absorbance and electrochemistry data of the investigated aluminum(III) porphyrins in CH_2_Cl_2_.

Sample	Absorption data^a^ *λ*_max,_ nm (logε)	Redox data^b^ *E*_1/2_ *vs* SCE	
		Oxidation	Reduction
AlC3P	397(4.57), 417 (5.46), 555 (3.99), 594 (3.54)	0.72, 0.88	−1.33, −1.76
AlC7P	398 (4.57), 417 (5.40), 555 (3.98), 594 (3.58)	0.76, 0.91	−1.34, −1.77
(AlC3P)_2_	396 (4.99), 416 (5.69), 555 (4.25), 594 (3.77)	0.76, 0.86	−1.34, −1.76
(AlC7P)_2_	396 (5.01), 417 (5.60), 557 (4.18), 595 (3.79)	0.75, 0.92	−1.34, −1.77
C_60_‐Im^c^	255 (5.25), 308 (4.73)	−	−0.66, −1.04, −1.56

^a^ in CH_2_Cl_2_

^b^ in CH_2_Cl_2_ with 0.1 M TBA·PF_6_

^c^ measured in *o*‐DCB, 0.1 M TBA·ClO_4_ [52].

**FIGURE 2:**
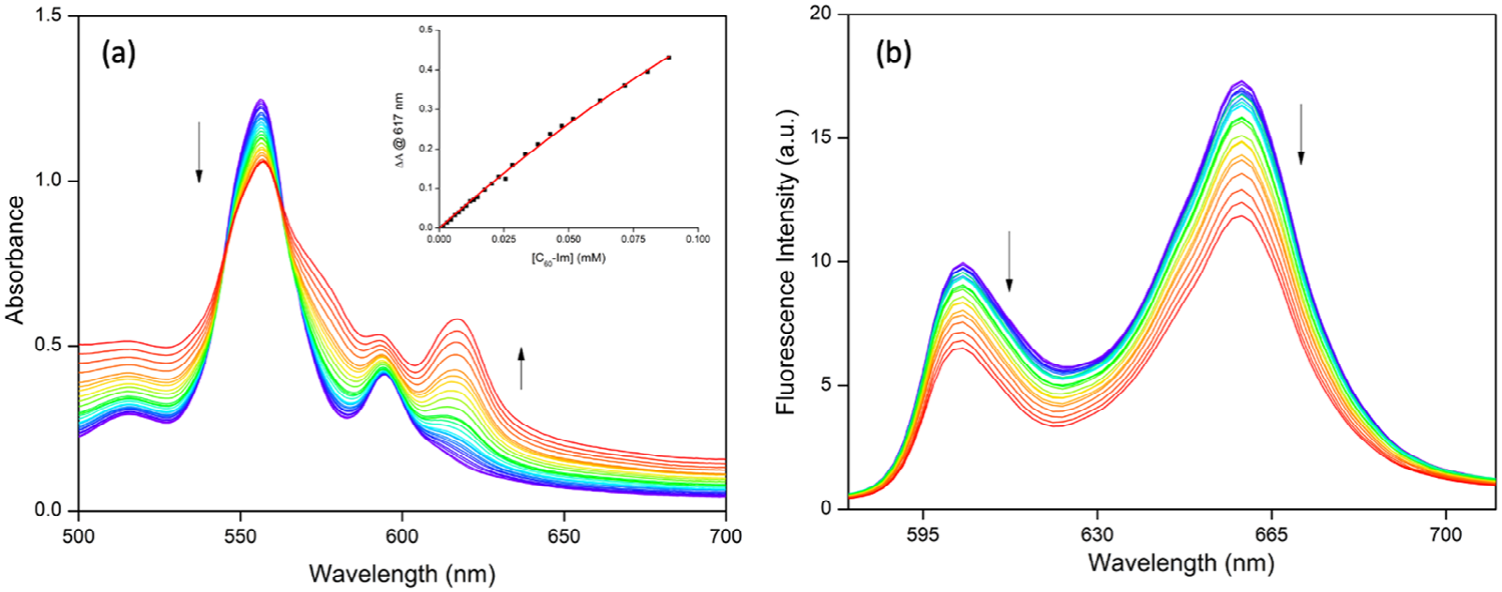
Absorption (a) and fluorescence (b) titrations of (AlC3P)_2_ *vs* C_60_‐Im in *o*‐DCB. C_60_‐Im was added up to 8.87 × 10^−5^ M in increments from 5 – 50 µL to a 1 mL (5 × 10^−5^ M) solution of (AlC3P)_2_. The inset shows the Thordarson curve fit. The isosbestic point 563 nm was utilized to excite the sample.

The self‐assembled supramolecular triads, C_60_‐Im→(AlC3P)_2_ and C_60_‐Im→(AlC7P)_2_, were obtained by mixing the constituent compounds, see Scheme 2. The formation of these systems was monitored by measuring the absorption and fluorescence spectra as a function of the ratio of the two components. Figure 2 shows the absorption titrations of (AlC3P)_2_ with C_60_‐Im in *o*‐DCB. The spectra were measured between 500 and 700 nm because both components absorb in this region, with the C_60_ absorption coefficients being slightly lower than those of the porphyrin. Upon addition of C_60_‐Im, the absorbance of the 555 nm band of the porphyrin decreases with an increase in new bands at 574 and 618 nm. These spectral changes can be rationalized as arising from a change in the planarity of the porphyrin ring as the Al center, which lies above the plane of the four pyrrole nitrogen atoms in the dyad, is drawn toward the coordinating ligand [54]. These spectral changes indicate the formation of self‐assembled triad C_60_‐Im→(AlC3P)_2_ with isosbestic points observed at 545 and 564 nm. Thordarson curve analysis, (fitting the data according to Equation (1)^21,39^ (Figure 2a, inset)) yields a binding constant *K* = 2.30 × 10^3^ M^−1^.

(1) $$\begin{array}{ccc} \Delta \;A_{617} & & =\frac{\varepsilon }{2}\;\lfloor \left(\left[\mathrm{Por}\right]+\left[A\right]+\frac{1}{K}\right)\\ & & -\sqrt{{\left(\left[\mathrm{Por}\right]+\left[A\right]+\frac{1}{K}\right)}^{2}-4\left[Por\right]\left[A\right]}\rfloor \end{array}$$

where Δ*A*_617_ is the absorbance change at 617 nm. [Por] (  = AlC3P, AlC7P, (AlC3P)_2_, or (AlC7P)_2_) is the total concentration of bound and unbound donor and is kept fixed, [A] (  = C_60_‐Im or Me‐Im) is the total concentration of the acceptor, which is varied, *K* is the binding constant, and *ε* is the molar absorptivity of the complex.

Similar spectral changes were observed in titrations of (AlC7P)_2_ with C_60_‐Im (Figure S7), confirming the formation of the triad C_60_‐Im→(AlC7P)_2_ with a similar binding constant of *K* = 3.67 × 10^3^ M^−1^. Additional titrations were carried out to establish the formation of the dyads C_60_‐Im→AlC3P (Figure S8a) and C_60_‐Im→AlC7P (Figure S9a). The observed spectral changes were analogous to those of the corresponding triads; thus, they establish the formation of dyads. Furthermore, titrations of (AlC3P)_2_ *vs* Me‐Im (Figure S10a), (AlC7P)_2_ *vs* Me‐Im (Figure S12a), AlC3P *vs* Me‐Im (Figure S10b), and AlC7P *vs* Me‐Im (Figure S12b) were performed in *o*‐DCB, (Me‐Im = 1‐methylimidazole). The spectral trends are consistent with their corresponding dyads and triads and were employed as controls in analyzing the excited state properties of the investigated systems. The corresponding binding constants, *K*, are reported in Table S1. The *K* values were found to be an order of magnitude smaller for C_60_‐Im binding compared to Me‐Im binding and were also an order of magnitude smaller than C_60_‐Im binding to other metalloporphyrins, such as zinc or tetraphenylaluminum(III) porphyrins. These results suggest overall weak coordination of C_60_‐Im to aluminum(III) porphyrin carrying meso‐alkyl substituents.

### 2.3 DFT calculations

**FIGURE 3:**
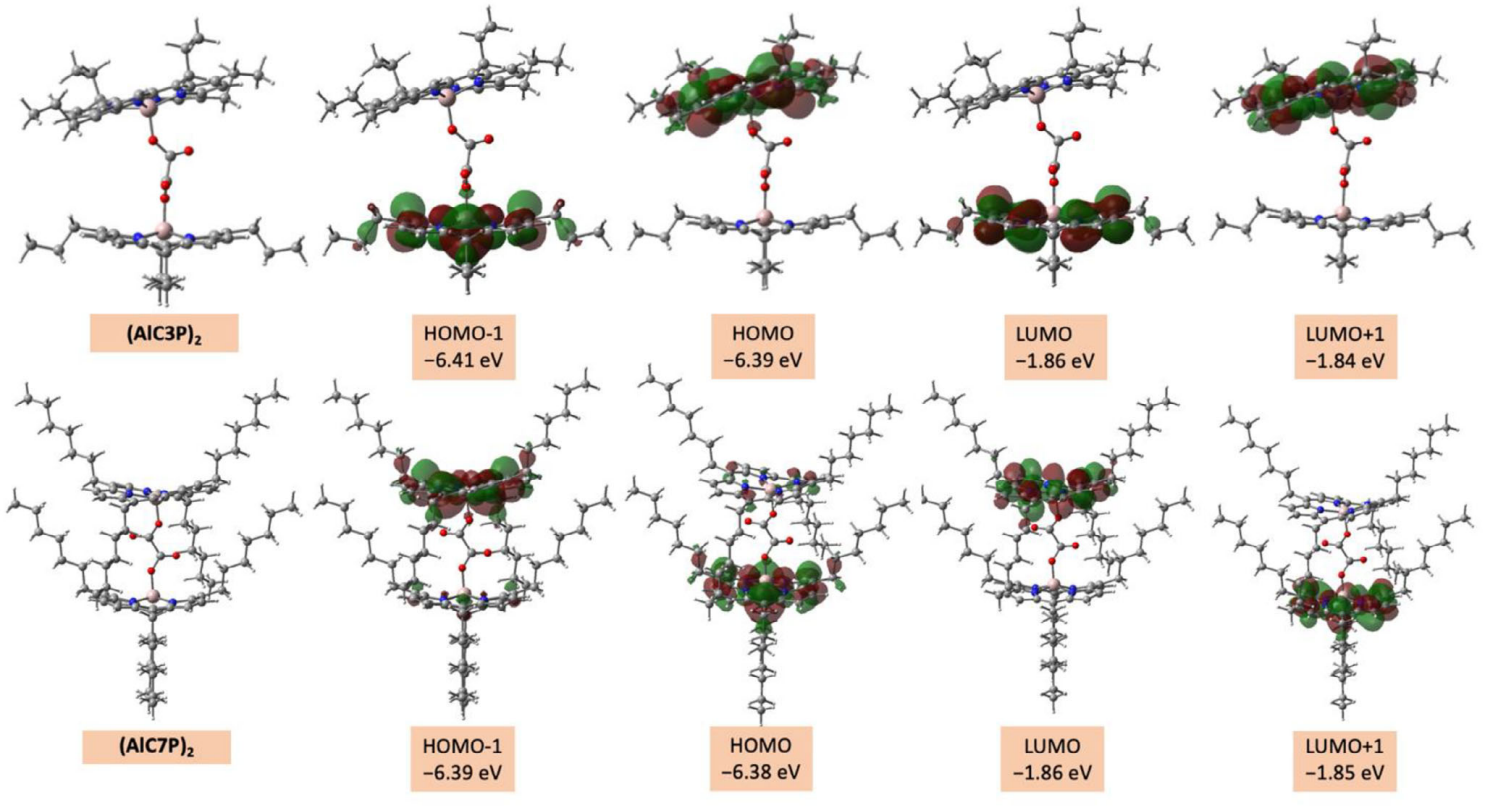
DFT calculated frontier orbitals of homodimer (AlC3P)_2_ and (AlC7P)_2_ systems.

The geometry and electronic structures of the homodimers were predicted by performing Density Functional Theory (DFT) calculations. Figure 3 shows optimized structures on a Born–Oppenheimer potential energy surface using the B3LYP functional and the 6–311G(d,p) basis set as parameterized in Gaussian 16 [72]. Following optimization, a single point energy calculation was performed using the Cam‐B3LYP functional and the 6–311G(d,p) basis set. As shown, the two porphyrin rings are aligned in a face‐to‐face arrangement with the four propyl/heptyl groups from each porphyrin pointing outside of the homodimer in a staggered fashion to minimize the steric repulsion. The Al center is found to lie out of the porphyrin plane and the center‐to‐center distance between the porphyrins is predicted to be 6.80 and 6.91 Å for (AlC3P)_2_ and (AlC7P)_2_, respectively. For the homodimers, the HOMO & HOMO–1 are predicted to be nearly degenerate as are the LUMO & LUMO+1. The HOMO‐1 and LUMO are localized on one of the porphyrin rings and the HOMO and LUMO+1 on the other. The estimated HOMO–LUMO gaps are 4.54 and 4.51 eV for (AlC3P)_2_ and (AlC7P)_2_, respectively. These values are larger than the experimental value 2.14 eV (estimated from the absorbance and fluorescence wavelengths of (AlC3P)_2_, see Figure S16). This difference is mostly due to the fact that the orbital energies are different in the excited state and ground state, that is, the HOMO‐LUMO gap in the ground state does not correspond exactly to the energy difference between the S_0_ and S_1_ states. These results complement the absorption spectra and confirm that the interaction between porphyrin rings of dimer molecules is very weak.

**FIGURE 4:**
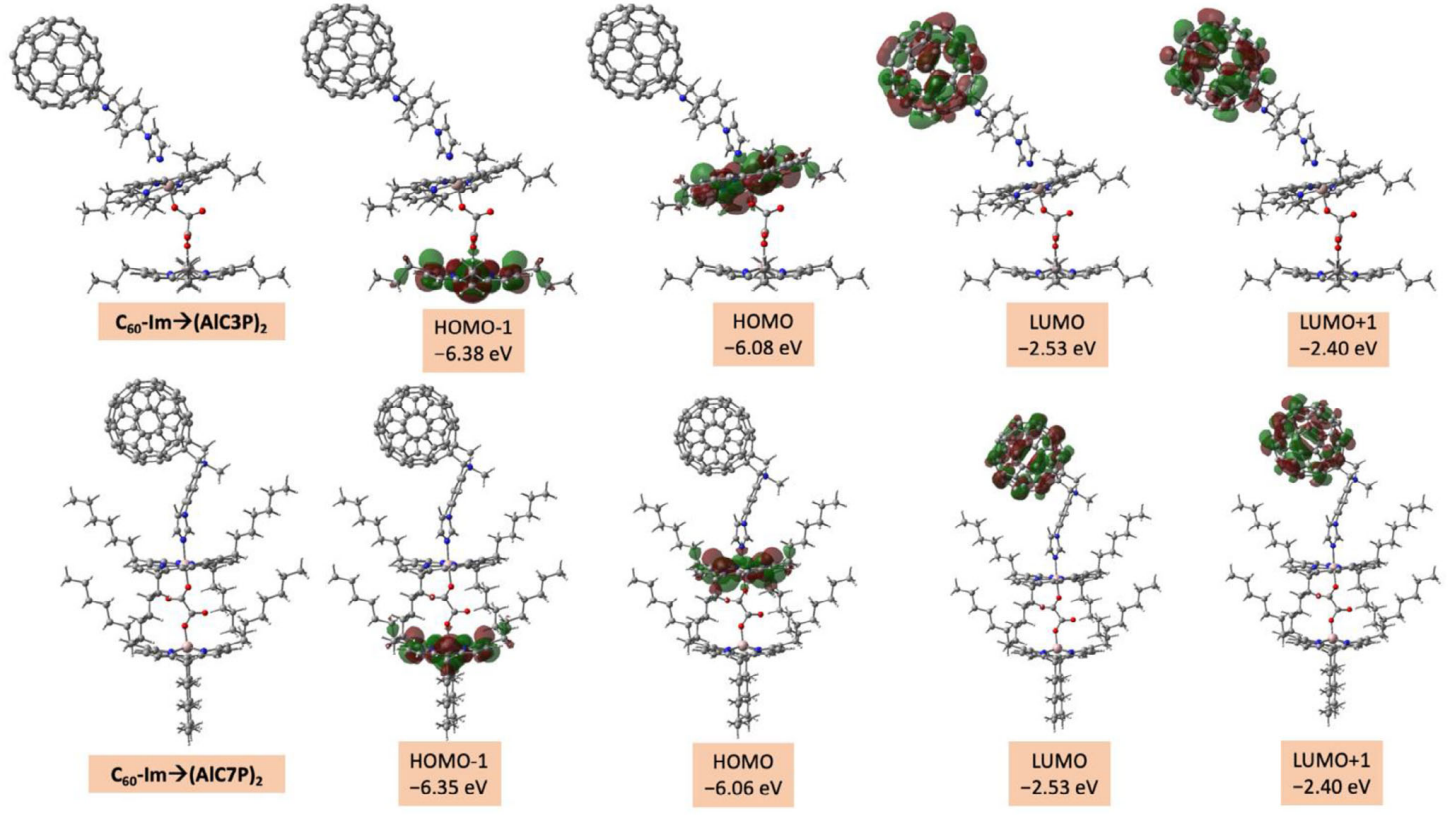
DFT calculated frontier orbitals of self‐assembled C_60_‐Im→(AlC3P)_2_ and C_60_‐Im→(AlC7P)_2_ systems.

In the case of the self‐assembled supramolecular triads, the HOMO, LUMO, and HOMO‐1 are mainly localized on the coordinated AlC3P/AlC7P, C_60_, and noncoordinated AlC3P/AlC7P, respectively, see Figure 4. The frontier orbital distribution indicates C_60_ acts as the primary electron acceptor, whereas the two porphryins of AlC3P/AlC7P act as sequential donors in the anticipated electron transfer process. The HOMO‐LUMO gap, which is roughly the energy needed to generate the charge‐separated states (C_60_)^•−^Im→(AlC3P)^•+^‐AlC3P or (C_60_)^•–^‐Im→(AlC7P)^•+^‐AlC7P, is estimated to be around 3.52 eV for both the supramolecular triads, again much higher than the experimentally predicted values. The plane‐to‐plane distance between two porphyrins was measured to be 6.74 and 6.90 Å for (AlC3P)_2_ and (AlC7P)_2_, respectively. It is important to note that the plane‐to‐plane distance is expected to decrease slightly in the triads compared to that of homodimers due to the hexavalent environment around the Al metal center.

### 2.4 Electrochemistry

**FIGURE 5:**
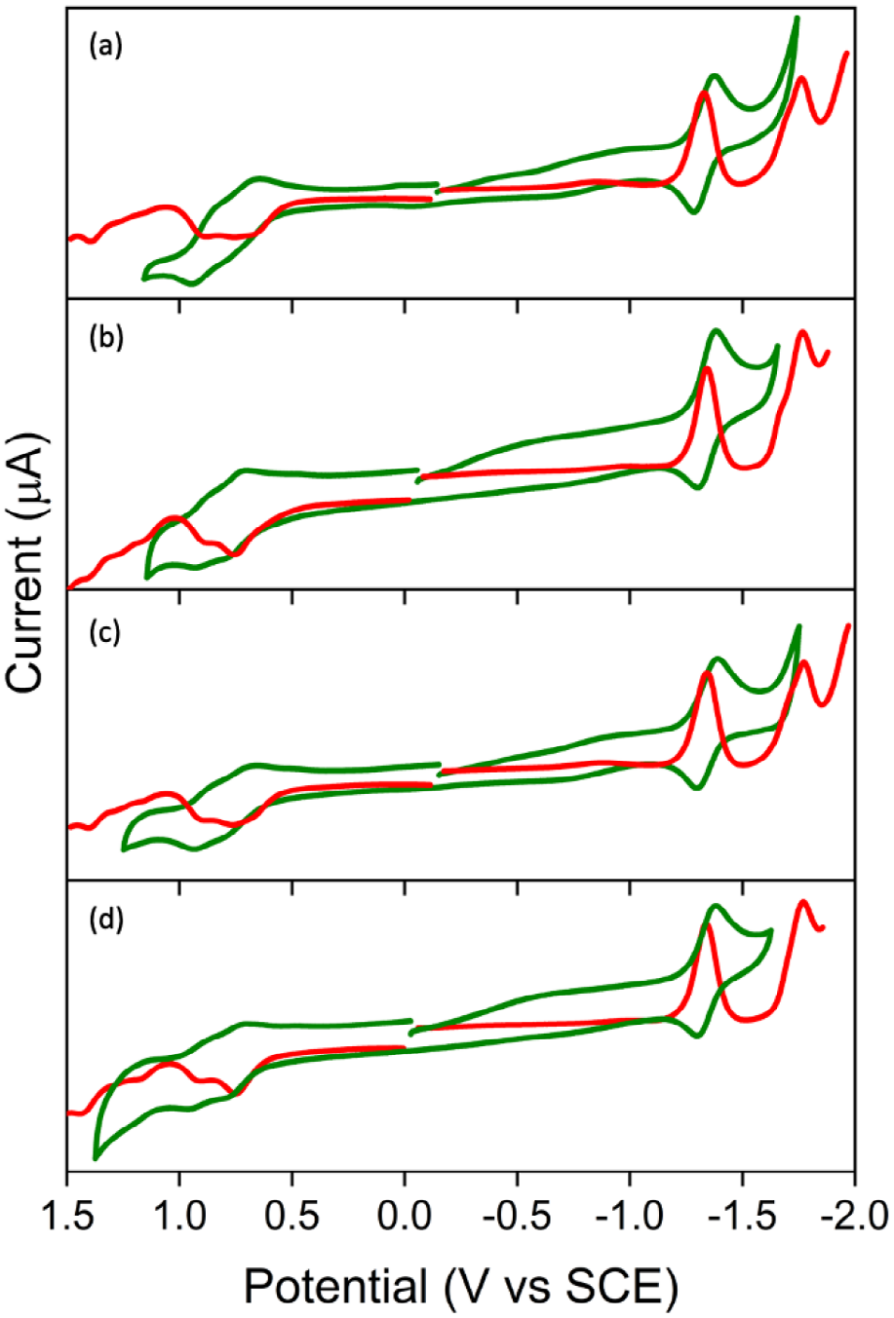
Cyclic (green) and differential pulse (red) voltammograms of the investigated aluminum(III) porphyrins in CH_2_Cl_2_ with 0.1 M TBA·PF_6_: (a) AlC3P, (b) AlC7P, (c) (AlC3P)_2_, and (d) (AlC7P)_2_. Scan Rate = 100 mV/sec, pulse period = 200 ms, pulse width = 50 ms, pulse amplitude = 50 mV.

Figure 5 shows the cyclic and differential voltammograms of the homodimer and its respective monomer porphyrins in CH_2_Cl_2_ with 0.1 M TBA·PF_6._ For the corresponding redox potentials, see Table 1. The cathodic scan of homodimers reveals two reversible one‐electron reduction processes between –1.34 and –1.77 V, corresponding to the successive addition of two consecutive electrons to the LUMO. Whereas the anodic scan revealed two oxidation processes between 0.75 and 0.92 V, corresponding to the consecutive removal of two electrons from the HOMO. The potentials of the homodimers are very similar to those of their monomer counterparts, which indicates that the electronic structure is not perturbed significantly in the homodimers. As expected, the length of the alkyl chain also does not influence the potentials. The C_60_‐Im potentials were adopted from the literature, which places the first reduction potential of C_60_‐Im at –0.66 V *vs* SCE [73].

### 2.5 Fluorescence Studies

**FIGURE 6:**
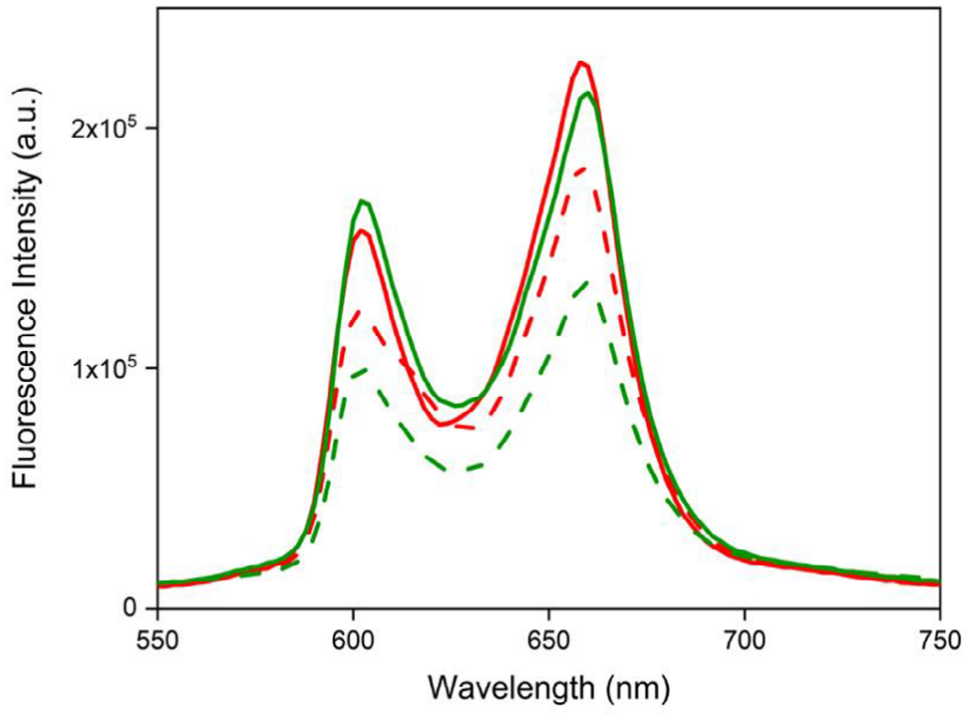
Fluorescence spectra of homodimers (AlC3P)_2_ (dashed red) and (AlC7P)_2_ (dashed green), and monomers AlC3P (solid red) and AlC7P (solid green) in o‐DCB. Wavelength excitation = 540 nm.

Steady‐state fluorescence measurements were carried out in *o*‐DCB solvent with excitation wavelength of 540 nm, see Figure 6. It is evident that the homodimer fluorescence intensities are slightly quenched (see %Q values in Table 2) compared to their reference monomers. The singlet state lifetimes are reported in Table 2, while the decay profiles are shown in Figure S14. The dimer lifetimes revealed little or no shortening of the lifetimes, suggesting the exciton coupling between porphyrins in the homodimers is too weak to influence the singlet state decay.

**TABLE 2:** Steady‐state fluorescence and lifetimes of the investigated porphyrins in *o*‐DCB.

Compound	Wavelength (%Q)^a^	Average *τ*, ns^b^	Goodness‐of‐fit (CHISQ)
AlC3P	602, 658	6.49	1.08
C_60_‐Im→AlC3P^c^	602, 659 (53%)	6.04	1.17
(AlC3P)_2_	602, 658 (10%)	6.57	1.13
C_60_‐Im→(AlC3P)_2_ ^c^	603, 659 (32%)	6.21	1.14
AlC7P	602, 660	6.49	1.06
C_60_‐Im→AlC7P^c^	603, 660 (32%)	6.59	1.17
(AlC7P)2	602, 660 (27%)	6.51	1.14
C_60_‐Im→(AlC7P)_2_ ^c^	602, 660 (42%)	6.45	1.04

^a^ Excitation wavelength = 540 nm (steady‐state studies) or isosbestic wavelength (titration studies)

^b^ Excitation wavelength = 560 nm and emission wavelength = 655 nm

^C^ C_60_‐Im:Porphyrin ratio = 3:1.

The steady‐state fluorescence titrations were carried out using solutions at a constant concentration of monomer or dimer and varying concentration of acceptor, analogous to absorption titrations. The solutions were excited at the isosbestic point wavelength, which was obtained from the corresponding absorption titrations. Figure 2b shows the fluorescence titrations of (AlC3P)_2_ *vs* C_60_‐Im in *o*‐DCB. For other studied systems, such data are shown in Figures S7–S9, S11 and S13. Upon addition of C_60_‐Im to (AlC3P)_2_, the porphyrin fluorescence exhibited some quenching, with no significant changes in wavelengths. By comparing these trends with control fluorescence titrations, AlC3P *vs* C_60_‐Im (Figure S8b), (AlC3P)_2_ *vs* Me‐Im (Figure S11a), and AlC3P *vs* Me‐Im (Figure S11b), it was reasonable to predict that the observed quenching could be due to oxidative electron transfer from ^1^(AlC3P)* to C_60_. This is because, the fluorescence quenching in the titrations of (AlC3P)_2_ (or AlC3P) vs C_60_‐Im is due to a decrease in free (AlC3P)_2_ dimer (or AlC3P) concentration as well as the formation of nonfluorescent triad C_60_‐Im→(AlC3P)_2_ (or dyad C_60_‐Im→AlC3P) due to the electron transfer process from AlC3P to C_60_ unit. However, in the case of (AlC3P)_2_ (or AlC3P) *vs* Me‐Im titrations, the peak positions and their intensity changes are due to the structural change that takes place during titrations, that is the Al center in AlC3P transitioning from a 5‐ to a coordinate system. As a consequence of this, the absorption and fluorescence peaks shift to longer wavelengths [54]. The spectral changes depicted in Figure S11 are combination of pentavalent (AlC3P)_2_ (with peaks at 601 and 659 nm) and hexavalent Me‐Im→(AlC3P)_2_ (with peaks at 625 and 687 nm) species. Similar conclusions can be reached from the titration of (AlC7P)_2_ *vs* C_60_‐Im (Figure S7b) that the C_60_‐Im→(AlC7P)_2_ is formed in solution. Based on the corresponding control titrations AlC7P *vs* C_60_‐Im (Figure S9b), (AlC7P)_2_ *vs* Me‐Im (Figure S13a), and AlC7P *vs* Me‐Im (Figure S13b), the additional quenching in C_60_‐Im→(AlC7P)_2_ is due to the oxidative electron transfer from ^1^(AlC7P)* to C_60_. These steady‐state studies confirm that electron transfer is possible from porphyrin to the C_60_ unit. However, it is not certain whether the resulting radical cation delocalizes over the two porphyrin π systems in the dimers. Such information could be retrieved through transient studies, which are discussed in the sections below.

### 2.6 Energetics

**FIGURE 7:**
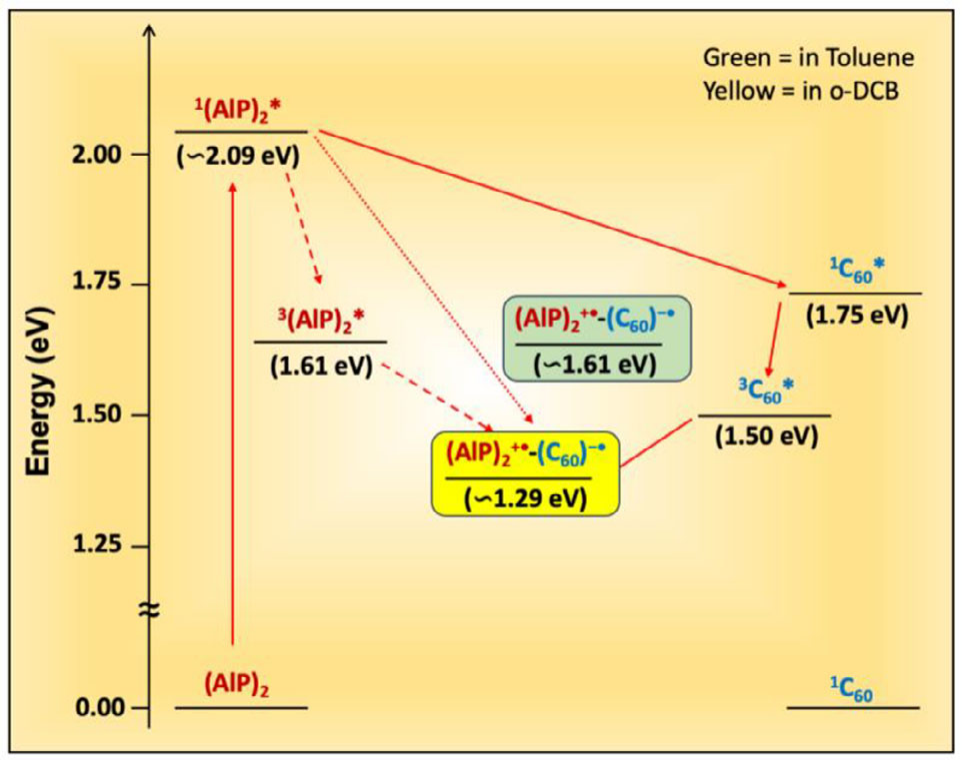
Energetics of investigated homodimers and triads. AlP represents both the AlC3P and AlC7P derivatives.

Figure 7 shows the energy levels diagram of the investigated compounds obtained from the redox potentials and optical data. The energies of the lowest excited singlet states (*E*_0−0_ = 2.09 eV) of homodimers were calculated by overlapping corresponding absorption and fluorescence spectra, see Figure S15. The *E*_0−0_ of C_60_ (  = 1.75 eV) and the lowest excited triplet states of (^3^(AlC3P/AlC7P)* = 1.61 eV) and C_60_ (  = 1.55 eV) have been taken from the literature [68, 73]. The energy of the charge‐separated states, E_CS_ (relative to the ground state), and the free‐energy changes for charge separation (*ΔG*_CS_) are estimated using the following Equations (2) and (3)

(2) $$E_{CS}=E_{1/2}^{ox}\left(D\right)-E_{1/2}^{red}\left(A\right)+G_{s}$$

Here, *G*_S_ is the ion‐pair stabilization and incorporates both the solvent‐dependent Coulomb energy change upon ion‐pair formation or recombination and the free energy of solvation of the ions, Equation (3)

(3) $$\begin{array}{cc} G_{S} & =e^{2}/\left(4\pi \varepsilon_{0}\right)\left[\left(1/\left(2R_{+}\right)+1/\left(2R_{-}\right)\;-\;1/R_{D-A}\right)\;1/\varepsilon_{S}\right.\\ & \;-\left.\left(1/\left(2R_{+}\right)+1/\left(2R_{-}\right)\right)\;1/\varepsilon_{R}\right] \end{array}$$

where R_+_, R_−_, and R_D‑A_ are donor radius (6.80 Å), acceptor radius (7.1 Å), and the center‐to‐center distance (13.48 Å) between donor and acceptor, respectively. ε_S_ is the dielectric constant of the solvent used for the photophysical studies, in this case, 9.93 and 2.38 for *o*‐DCB and toluene, respectively. ε_R_ is the dielectric constant of the solvent used for measuring the redox potentials, in this case 8.93 for CH_2_Cl_2_. Using the radii from the DFT calculations, *G*_S_ values of −0.13 and 0.19 eV are obtained for (C_60_)^•–^‐Im→(AlC3P)_2_^•+^ / (C_60_)^•–^‐Im→(AlC7P)_2_^•+^ in *o*‐DCB and Toluene, respectively. The calculated free‐energy level diagrams suggest that the oxidative electron transfer occurs from the excited singlet state of AlC3P or AlC7P to the C_60_ unit. Alternatively, singlet‐singlet energy transfer from ^1^AlP* to C_60_ is also thermodynamically feasible.

### 2.7 Transient Absorption Spectroscopy

To obtain additional evidence for the anticipated excited state dynamics and energy/electron transfer processes upon photoexcitation of the supramolecular monomers, dimers, and triads, femtosecond and nanosecond transient spectroscopic measurements were performed in *o*‐DCB at an excitation wavelength of 415 nm (Soret band).

**FIGURE 8:**
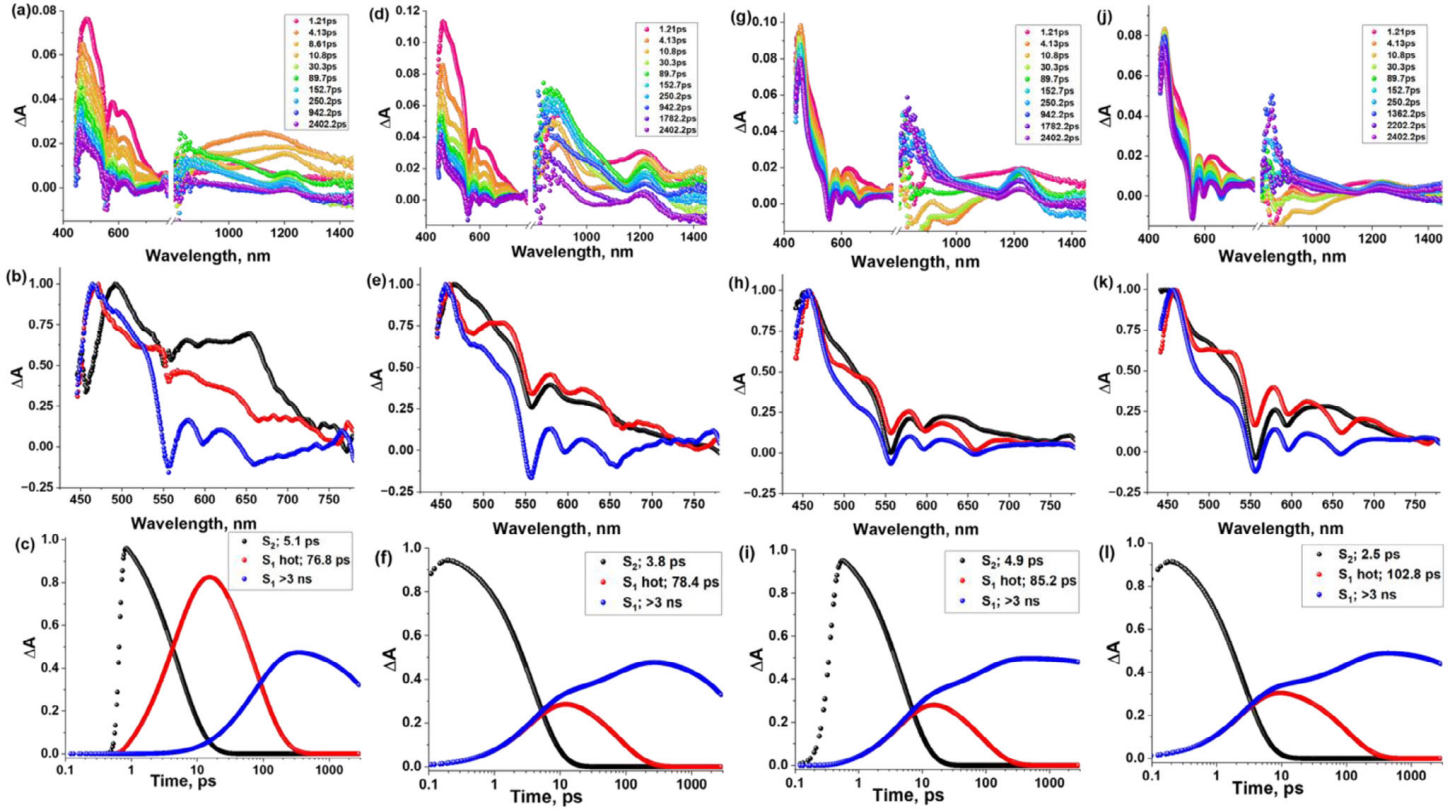
Femtosecond transient absorption spectra at the indicated delay times (λ_ex_ = 415 nm) of (a) AlC3P, (d) (AlC3P)_2_, (g) AlC7P, (j) (AlC7P)_2_ in solvent *o*‐DCB. Their respective decay‐associated spectra (b, e, h, and k) and population curves (c, f, i, and l) are shown in rows 2 and 3.

Figure 8 shows femtosecond transient absorption spectra at the indicated delay times for AlC3P, AlC7P, (AlC3P)_2_, and (AlC7P)_2_. The transient spectral features of the monomers and their respective dimers have similar patterns with subtle differences. In the spectra of AlC3P and dimer (AlC3P)_2_ in the first few ps after the laser flash (see e.g. the spectrum at ∼1.21 ps in Figure 8a, d), excited‐state absorption (ESA) peaks at 465, 580, 620, 710, 770 nm, and 1205 nm of the excited singlet state are observed. The 1205 nm peak has been attributed to the singlet‐singlet transition of the ^1^AlP* entity of the monomer and dimer [67]. Additionally, two negative peaks at 555 nm and 605 nm were observed that correspond to ground‐state bleaching (GSB) and stimulated emission (SE), respectively. The decay of the positive peaks and recovery of the negative peak are accompanied by the appearance of new peaks at 500 and 840 nm, corresponding to ^3^AlC3P^*^ formed *via* intersystem crossing (ISC). Similarly, in the case of AlC7P and dimer (AlC7P)_2_, early time scale transient spectra show the ESA peaks at 460, 580, 620,720, and 770 nm and 1205 nm with negative peaks at 555 nm corresponding to GSB and 605 nm corresponding to SE. The decay of these ESA peaks as the triplet state is populated by ISC gives rise to new peaks at 500 and 840 nm. Further, target analysis was performed using GloTarAn software to obtain the decay‐associated spectra (DAS), and population kinetics. (shown in Figure 8). A sequential kinetic scheme with three components corresponding to the S_2_, S_1_hot, and S_1_ excited states was used, and their lifetimes are reported in the legends of their respective population curves in Figure 8c,f,i,l. The S_1_ lifetime was obtained as >3 ns, which is consistent with the singlet state lifetime obtained by the TCSPC studies (see Table 2), and a similar observation can be concluded from the 1205 nm peak (corresponding to singlet‐singlet ESA) that didn't decay completely within the 3 ns detection limit of the femtosecond instrument (see 1205 nm peak at longer delay time) for all four compounds. To obtain the lifetime of the T_1_ state, nanosecond transient absorption spectra were also recorded. We obtained the triplet state lifetimes as 1.45, 1.14, 9.247, and 2.14 µs in AlC3P, (AlC3P)_2_, AlC7P, and (AlC7P)_2_, respectively. (see Figure S16).

**FIGURE 9:**
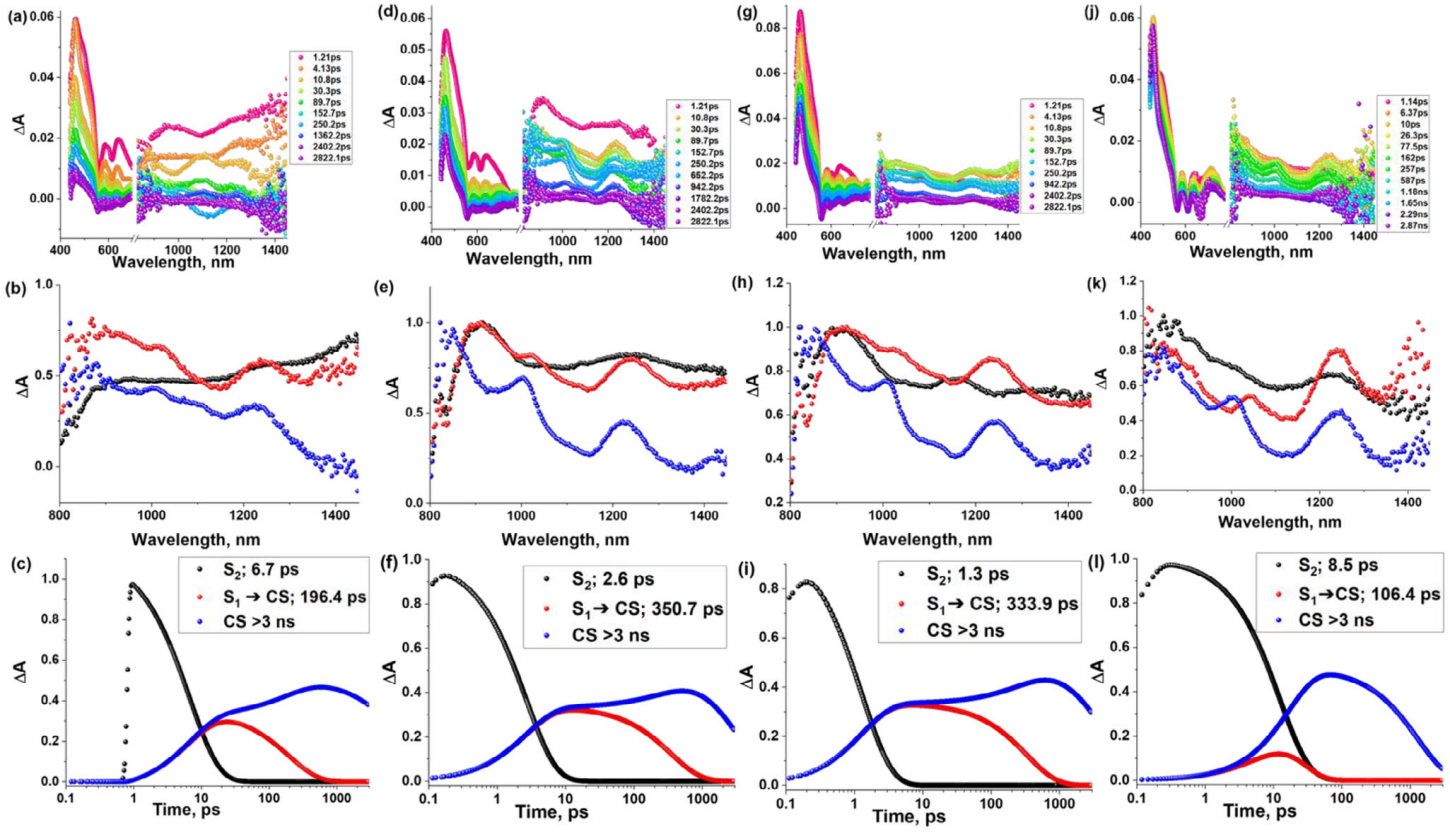
Femtosecond transient absorption spectra at the indicated delay times (*λ*_ex_ = 415 nm) of (a) C_60_‐Im→AlC3P, (d) C_60_‐Im→(AlC3P)_2_, (g) C_60_‐Im→AlC7P, (j) C_60_‐Im→(AlC7P)_2_ in solvent *o*‐DCB. Their respective decay‐associated spectra and population curves are shown in rows 2 and 3.

To obtain the evidence for the presence of a charge separation state in the above‐described compounds with C_60_, the transient absorption spectra of dyads C_60_‐Im→AlC3P, C_60_‐Im→AlC7P, and triads C_60_‐Im→(AlC3P)_2_, C_60_‐Im→(AlC7P)_2_ were recorded as shown in Figure 9. Careful inspection of these spectra shows the presence of the characteristic anion radical peak at 1020 nm for C_60_^•^ **^–^** in both the dyads C_60_‐Im→AlC3P, C_60_‐Im→AlC7P and triads C_60_‐Im→(AlC3P)_2_, C_60_‐Im→(AlC7P)_2_. This characteristic peak is weak and broad in the case of C_60_‐Im→AlC3P, which indicates a lower population of charge‐separated (CS) state. A possible reason for the lower yield in this case could be fast energy transfer from ^1^AlP* to C_60_. By comparing the transient spectral peaks with their corresponding oxidation titration spectra shown in Figure S17, we assign the initial peaks formed at around 600, 730, and 810 nm to the porphyrin cation radical species. Target analysis was performed using a three‐component scheme involving the S_2,_ S_1_, and CS states. The lifetimes of these different energy states are shown in the legends of their respective population curves (see Figure 9c, f, i l). As the CS population is slow in this system, the S_1_ state lifetime may have some contribution from the CS state, which is the reason this lifetime does not align with the TCSPC lifetime, but the decay of the 1250 nm peak strongly supports that S_1_ state lifetime in the ns range that aligns with TCSPC data (partial or overlapped deconvolution). The lifetime of CS state was obtained as >3 ns, hence, nanosecond transient absorption spectra were recorded (see Figure S18), and the decay profile of C_60_ anion peak at 1020 nm is shown in Figures S18c and S18f. These signals were not very strong, which is why the obtained spectra are very noisy; however, they provided a reasonable decay profile of the anionic peak to secure the lifetime of the charge‐separated states. However, the uncertainty in the lifetimes is too large to allow the small differences between the values for the different triads to be interpreted reliably in terms of structure‐dynamics relations.

Rate constants of the formation of the CS state (*k*_CS_) and charge recombination (*k*_CR_) state, along with the average lifetime of radical ion pair (*τ*_RIP_) in dyads and triads, are presented in Table 3. The C_60_ anion peak at 1020 nm was used to calculate the rate constant, and its decay profile is shown in Figure S18. The rise time of 1020 nm peak (τ_rise_) provides information about the rate constant of charge separation state, where *k*_CS_ = 1/*τ*_rise,_ while the average decay time of 1020 nm peak (*τ*_RIP_) corresponds to the average lifetime of the radical ion pair formed and suggests the rate constant for charge recombination as *k*_CR_ = 1/*τ*_RIP_.

**TABLE 3:** Rate constants *(k*_CS_ and *k*_CR_) and lifetimes (*τ*_rise_ and *τ*_RIP_) for dyads and triads in o‐DCB.

Compound	*τ*_rise_ (ps), *k*_CS_ × 10^10^ (s^−1^)	*k*_CR_ × 10^6^ (s^−1^)	*τ*_RIP_ (average in µs)
C_60_‐Im→AlC3P	1.08, 92.6	0.23	4.38
C_60_‐Im→(AlC3P)_2_	1.17, 85.5	0.29	3.37
C_60_‐Im→AlC7P	1.24, 80.6	0.42	2.36
C_60_‐Im→(AlC7P)_2_	0.93, 107.5	0.26	3.76

### 2.8 Transient EPR Spectroscopy

**FIGURE 10:**
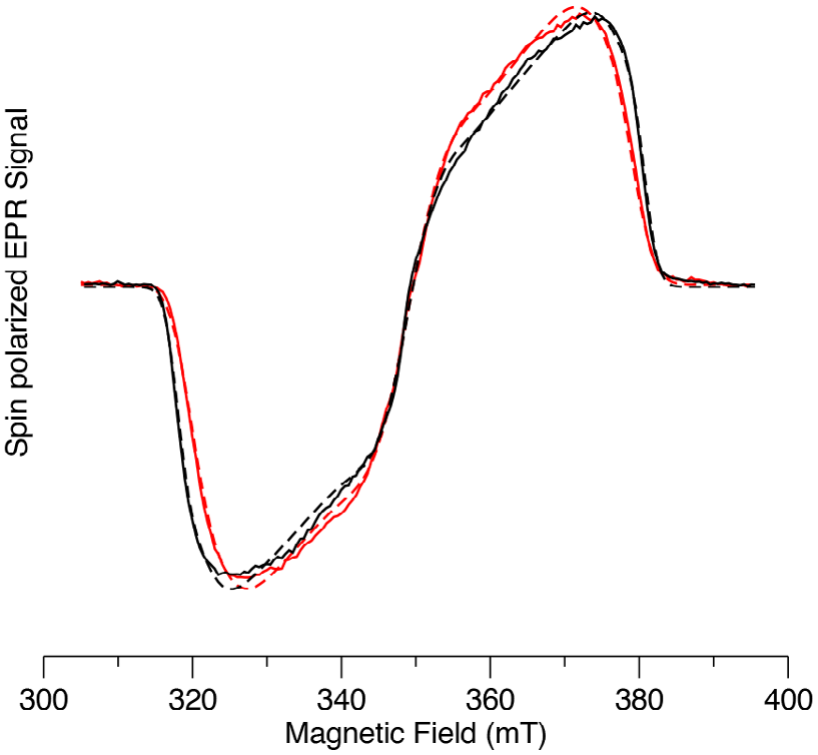
Spin polarized transient EPR spectra of AlC3P (black) and (AlC3P)_2_ (red) in toluene at 80 K. The dashed curves are simulations of the measured spectra. The spectra have been normalized to the same amplitude and a microwave frequency of 9.794 GHz.

Figure 10 shows a comparison of the spin‐polarized transient electron paramagnetic resonance (TREPR) spectra of AlC3P and (AlC3P)_2_ in toluene at 80 K. The corresponding spectra of AlC7P and (AlC7P)_2_ are shown in Figure S19. The spectra of the monomers (black) are typical of the triplet state of Al porphyrins. The broad emission/absorption (E/A) pattern arises from selective population of the triplet sublevels according to their *x* and *y* character, and the shape of the spectrum shows that the rhombicity of the zero‐field splitting (ZFS) tensor is large. The values of the ZFS parameters and relative sublevel populations are given in Table 4 and have been obtained from simulations of the spectra (dashed curves in Figure 10). The ZFS parameter D determines the overall width of the spectrum and describes the strength of the spin‐spin interaction between the unpaired electrons in the triplet state. As can be seen in Figures 10 and S19 and Table 4, the spectra of the dimers and monomers are all very similar, and the ZFS parameters are all almost the same. Thus, in the dimers, it appears that the triplet excitation is localized on one of the constituent monomers. It is also possible that triplet hopping between the two monomers occurs, provided that no averaging on the ZFS takes place. This happens when the hopping rate is much less than the ZFS in frequency units or if the orientation of the principal axes of the ZFS tensor relative to the magnetic field is the same for both monomers. Closer inspection of Figure 10 and Table 4 shows that the value of the ZFS parameter D is slightly smaller in (AlC3P)_2_ (red spectrum) than in AlC3P (black spectrum). This suggests that a slightly greater degree of delocalization or a small amount of averaging due to triplet hopping occurs in the dimer. However, the size of the effect is very small.

**TABLE 4:** Triplet state zero‐field splitting parameters and relative ISC population rates at 80 K in toluene.

Complex	Triplet State	D (MHz)	E (MHz)	(*p*_x_−*p*_y_):(*p*_y_–*p*_z_)
AlC3P	^3^AlP	880	236	0.07 : 0.46
(AlC3P)_2_	^3^AlP	837	218	0.04 : 0.48
AlC7P	^3^AlP	884	211	0.04 : 0.48
(AlC7P)_2_	^3^AlP	874	217	0.04 : 0.48
C_60_‐Im→AlC3P	^3^AlP	857	213	−0.86 : 0.72
	^3^C_60_	−250	−23	−0.79 : 0.90
C_60_‐Im→AlC3P	^3^AlP	874	179	−0.51 : 0.75
	^3^C_60_	−260	−26	−0.70 : 0.85
C_60_‐Im	^3^C_60_	−260	−32	−0.18 : 0.59

**FIGURE 11:**
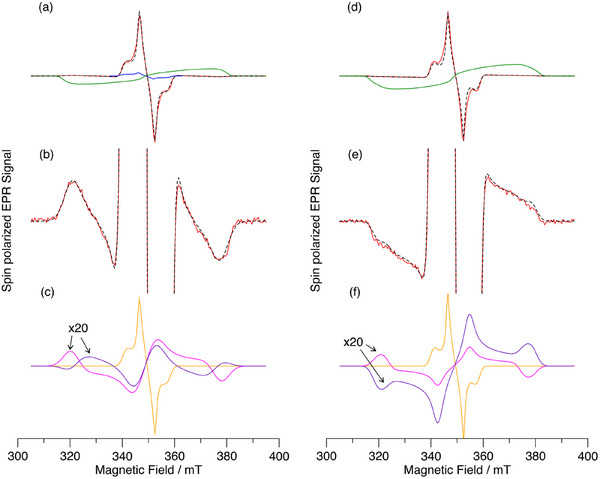
Experimental and simulated spin polarized transient EPR spectra of C_60_‐Im→AlC3P and C_60_‐Im→AlC7P and the reference compounds C_60_‐Im, AlC3P and AlC7P in toluene at 80 K. (a) Experimental spectra of C_60_‐Im→AlC3P (red), AlC3P (green) and C_60_‐Im (blue) and simulation of the C_60_‐Im→AlC3P spectrum (dashed black) (b) Experimental (red) and simulated (dashed black) spectra of C_60_‐Im→AlC3P on an expanded scale to show the ^3^AlPor signals. (c) Contributions to the simulated spectrum of C_60_‐Im→AlC3P: ^3^C_60_ formed by ISC (orange), ^3^AlP formed by ISC (purple), and radical pair recombination (magenta). The ^3^AlP spectra have been multiplied by a factor of 20. (d) Experimental spectra of C_60_‐Im→AlC7P (red) and AlC7P (green). Simulation of the C_60_‐Im→AlC7P spectrum (dashed black) (e) Experimental (red) and simulated (dashed black) spectra of C_60_‐Im→AlC7P on an expanded scale to show the ^3^AlP signals. (f) Contributions to the simulated spectrum of C_60_‐Im→AlC7P: ^3^C_60_ formed by ISC (orange), ^3^AlP formed by ISC (purple), and radical pair recombination (magenta). The ^3^AlP spectra have been multiplied by a factor of 20.

Spin‐polarized TREPR spectra of dyads C_60_‐Im→AlC3P and C_60_‐Im→AlC7P at 80 K in toluene are shown in Figure 11a, d, respectively (red spectra). The TREPR spectra of AlC3P and AlC7P (green spectra) and C_60‐_‐Im (blue spectrum) are also shown in Figure 11a, d for comparison. The spectra of the dyads (Figure 11a, b, red) have a very strong contribution from the C_60_ triplet state (^3^C_60_), which is much more intense than the corresponding spectrum of C_60_‐Im (Figures 11a, blue and S20) at the same concentration. A very weak contribution from the porphyrin triplet state (^3^AlP) is also observed (Figure 11b, e), with a different polarization pattern than that obtained without C_60_ coordination (Figure 11a, d, green spectra). The greater intensity of the C_60_ triplet state spectrum and weaker porphyrin triplet state contribution imply that energy transfer from AlP to C_60_ occurs, especially in nonpolar toluene at low temperature. The change in the polarization of the weak porphyrin triplet spectrum implies that the pathway by which it is populated is different when C_60_ is coordinated. The absorptive polarization on the low‐field end of the porphyrin triplet state spectrum is expected to result in radical pair recombination to the triplet state. We have simulated the spectra assuming that they are the sum of three contributions: i) the C_60_ triplet state populated by ISC, ii) the porphyrin triplet state populated by ISC, and iii) the porphyrin triplet state populated by radical pair recombination (note that the energy of the radical ion‐pair is expected to be higher than that of the triplet state in the frozen glass due to the lack of solvent reorganization., see Figure 7). The resulting fitted spectra are the black dashed curves in Figure 11a, b,d, and e. As can be seen, excellent agreement with the experimental spectrum is obtained. The three contributions are plotted separately in Figure 11c, f, and the parameters used in the simulation are given in Table S2. In both dyads, the C_60_ triplet state formed by ISC dominates. The relative population rates of the zero‐field levels of the ^3^C_60_ contribution in the dyads are different than those in the reference compound C_60_‐Im (compare Figures 11 and S20). We speculate that this is due to a difference in the excited state dynamics in the bound C_60_ compared to the unbound reference compound. It is also possible that some triplet‐triplet energy transfer from ^3^AlP to ^3^C_60_ occurs in the dyad. The simulations suggest that the much weaker contribution from the AlP triplet state is because it has a larger contribution from radical pair recombination in C_60_‐Im→AlC3P than in C_60_‐Im→AlC7P (see Table S2).

**FIGURE 12:**
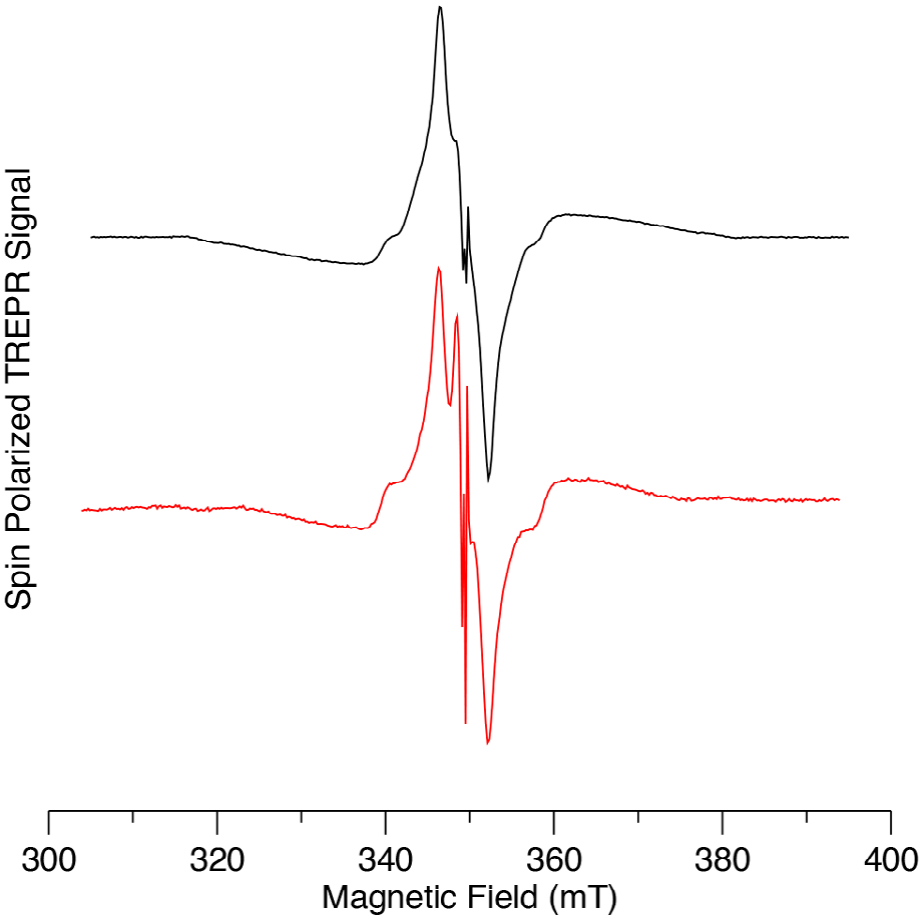
Spin polarized transient EPR spectra of C_60_‐Im→(AlC3P)_2_ (black) and C_60_‐Im→(AlC7P)_2_ (red) in toluene at 80 K. The spectra have been normalized to the same maximum amplitude.

Figure 12 shows the corresponding spectra for the two dimers with C_60_ coordinated to them. The spectra are like those of the monomers but show some important differences. The most striking of these differences is the presence of narrow peaks in the center of the spectrum, which  likely arise from a radical pair. The relative intensity of the ^3^C_60_ contribution is also weaker than with the monomers, suggesting that less energy transfer from AlP to C_60_ occurs. The ^3^AlP contribution exhibits an emission/absorption pattern indicative of its formation primarily through intersystem crossing. Together, these results suggest that in the dimer complexes, there is a higher yield of charge separation and a longer lifetime of the charge‐separated state, consistent with stabilization of the radical pair.

**FIGURE 13:**
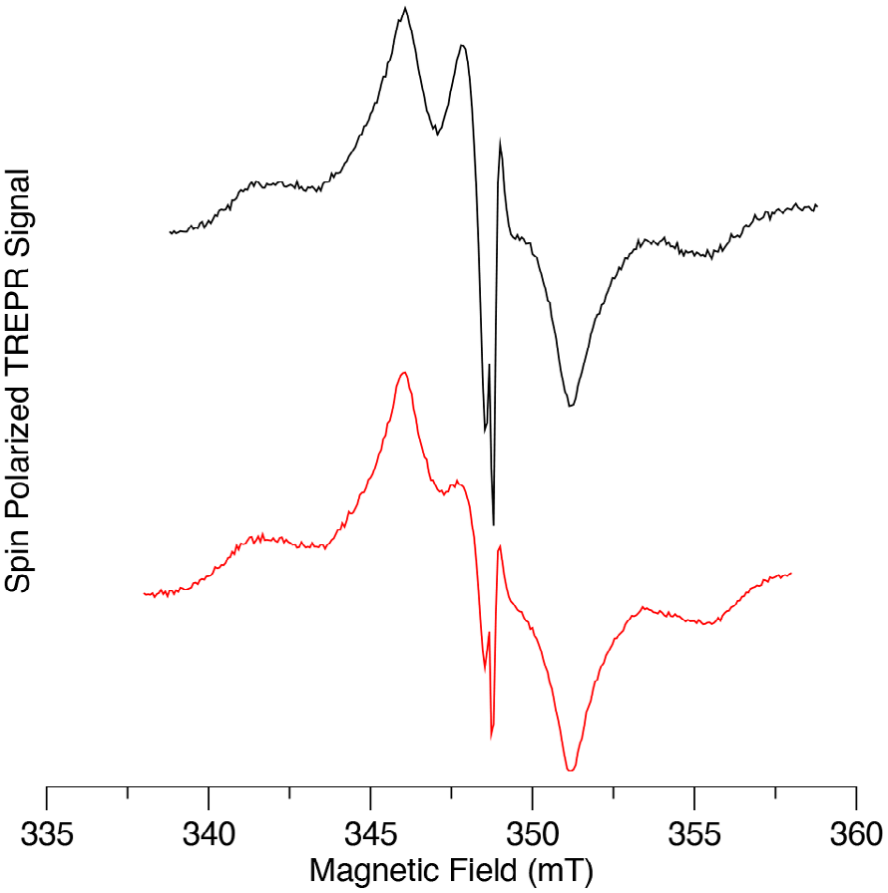
Spin polarized transient EPR spectra of C_60_‐Im→AlC3P (black) and C_60_‐Im→AlC7P (red) in toluene at 180 K. The spectra have been normalized to the same maximum amplitude.

The observation of charge separation in the glass phase of toluene at 80 K is unusual for donor‐acceptor complexes, as the solvent is nonpolar and reorganization stabilization is not possible. This suggests that at higher temperatures and/or in more polar environments, a greater yield can be expected. Thus, we have also measured the complexes at 180 K, just below the glass transition temperature of toluene. Figure 13 shows the TREPR spectra of the two monomers with coordinated C_60_ under these conditions. As can be seen, both complexes exhibit narrow peaks at the center of the spectrum due to a radical pair, with a comparatively weak contribution from the C_60_ triplet state. Note that the sweep range of the spectrum is narrower than that used at 80 K because the contribution from the AlPor triplet state was too weak to be measured reliably. The comparison of Figures 11 and 13 shows that raising the temperature from 80 to 180 K leads to a higher yield and longer lifetime of the charge separation in the AlPor monomer C_60_ complexes.

**FIGURE 14:**
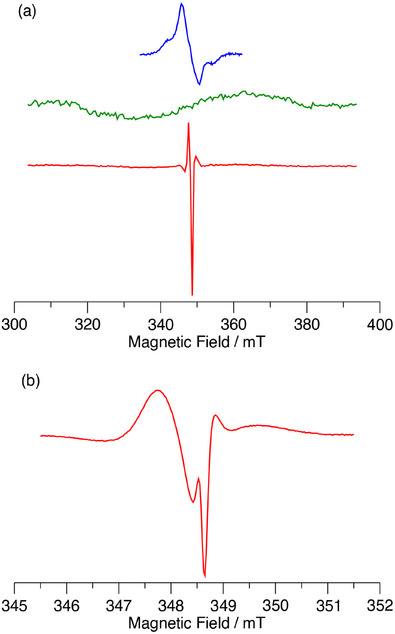
Spin polarized transient EPR spectra of (a) C_60_‐Im (blue), (AlC3P)_2_ (green), and C_60_‐Im→(AlC3P)_2_ (red) and (b) C_60_‐Im→(AlC3P)_2_ on a narrow field range at 180 K in toluene.

For the dimer complex C_60_‐Im→(AlC3P)_2_, the effect of the increase in temperature is even more dramatic, as illustrated in Figure 14. In this case, only the radical pair peaks are observed, and there is almost no signal from ^3^C_60_ of ^3^AlP, as can be seen from the comparison of the TREPR spectrum C_60_‐Im→(AlC3P)_2_ with those of the two reference compounds in Figure 14a. Careful examination of the wings of the spectrum reveals extremely weak contributions from these two species. Simulation of the radical pair spectrum is complicated by the fact that some motional averaging likely occurs in the soft glass, and the exchange and dipolar coupling between the radicals appear to be similar in magnitude. Because the dipolar coupling is orientation dependent and changes sign, it means that for some orientations the coupling between the two spins of the radical pair is weak, while it is much stronger for others. This makes the spectrum very sensitive to the values of the two coupling constants and the geometry of the radical pair. Thus, we have not attempted a quantitative analysis of the radical pair spectrum. However, the emissive polarization on the low field end of the spectrum is consistent with the formation of the radical pair by singlet electron transfer if the dipolar coupling dominates and is negative. Similar results are also observed for the C_60_‐Im→(AlC7P)_2_ (Figure S21) except that slightly more of the two triplet states are formed.

To increase the reorganization stabilization, we also measured the samples at room temperature in the *o*‐DCB and the liquid crystal 5CB (Figure S22). In 5CB, the spectra are similar to those measured at 180 K in toluene, except that a greater contribution from the C_60_ triplet state is observed. This may be due to the binding of the nitrile group of 5CB to the Al center. The binding constant for the imidazole group of C_60_‐Im should be much higher than that of the nitrile group, but the much higher concentration of the solvent probably leads to replacement of the C_60_ acceptor by a solvent molecule in a fraction of the complexes. In *o*‐DCB, a weak E/A pattern of the radical pair is observed. If the triplet states are present, the fast isotropic motion in this solvent should average the multiplet polarization, leaving only weak net polarization. The spectrum (Figure S22 bottom) does not show any net polarization, suggesting that the triplet states are not populated. However, the intensity of the radical pair signal is weak, which implies that a significant fraction of the excited complexes relaxes rapidly to the ground state. Because this fraction does not contribute to the TREPR signal, the pathway of relaxation remains unclear.

Obtaining the radical pair lifetime from the TREPR data is difficult because the time dependence of the signals is governed by a combination of T1 relaxation, T2 relaxation, the motion of the magnetization in the microwave field, and the lifetime of the radical pair. Deconvoluting these various effects is challenging. However, the lifetime of the spin‐polarized signal gives a lower limit for the RP lifetime. At room temperature in DCB we obtain a lifetime of 4µs for the TREPR signal decay of C_60_‐Im→(AlC3P)_2_ in DCB. This is very close to the lifetimes obtained from the ns TA measurements (Table 3), suggesting the TREPR signals decay mostly because of charge recombination under these conditions. The RP signal from C_60_‐Im→(AlC3P)_2_ in toluene is still visible at 8µs after the laser flash at 180 K and 16 µs at 80 K. Thus, the RP lifetime increases significantly with decreasing temperature.

## 3 Conclusion

An aluminum homodimer‐based series was synthesized and investigated in the presence and absence of an electron‐deficient C_60_‐Im moiety. The transient optical data show that when C_60_‐Im is coordinated to the Al(III) center, charge separation occurs in both the dyads and triads. Although the excitonic coupling between the two porphyrins is weak, the dimeric structure of the electron donor increases the lifetime of the charge separation in the triads compared to the dyads. Time‐resolved EPR and transient absorption studies show that in the dyads, the charge‐separated state is formed from the excited singlet state of the porphyrins and has a lifetime of ∼4 µs at room temperature. At lower temperatures, the TREPR data show that the yield of the charge‐separated state decreases as the solvent mobility decreases, while the charge‐separation lifetime increases. This system opens the door to further studies in which the influence of porphyrin coupling on the yield and lifetime of charge separation can be investigated by varying the bridging group structure.

## Conflicts of Interest

The authors declare no conflicts of interest.

## Supporting information



Details of synthesis, photophysical, electrochemical, and DFT methods; ESI mass and NMR spectra; Absorption and fluorescence titrations; Binding constants; Density difference maps; TCSPC decay profiles, *E*_0‐0_ spectra; Nanosecond transient absorption spectra; Chemical oxidation spectra; TREPR spectra and corresponding simulation data Tables.

## Data Availability

The data that supports the findings of this study are available in the supplementary material of this article
