# Supplementary material for: Mimicking The Photosynthetic Special Pair With Aluminum(III) Porphyrin Homodimer – Fullerene (C60) Supramolecular Assemblies Capable of Long‐Lived Charge Separation
Source: Chemistry. 2026 Mar 13;32(18):e70853. doi: 10.1002/chem.70853 (PMC13174913; doi:10.1002/chem.70853)
Supplement: Supplementary file 1 — Details of synthesis, photophysical, electrochemical, and DFT methods; ESI mass and NMR spectra; Absorption and fluorescence titrations; Binding constants; Density difference maps; TCSPC decay profiles, E 0‐0 spectra; Nanosecond transient absorption spectra; Chemical oxidation spectra; TREPR spectra and corresponding simulation data Tables. [file CHEM-32-e70853-s001.docx]

**Supporting Information**

Mimicking Photosynthetic Special Pair Capable of Charge Separation with Long-Lived Lifetimes in Aluminum(III) Porphyrin Homodimer – Fullerene (C_60_) Supramolecular Assemblies

### *Stefan Charon,^a^ Jatan K. Sharma,^b^ Niloofar Zarrabi, ^a^ Peyton Ellis,^a^ Paul A. Karr,^c^ Art van der Est,^d,*^ Francis D’Souza,^b,*^ Prashanth K. Poddutoori ^a,*^*

*^a^Department of Chemistry & Biochemistry*, University of Minnesota Duluth, 1038 University Drive, Duluth, Minnesota 55812, USA. *^b^*Department of Chemistry, University of North Texas, 1155 Union Circle, # 305070, Denton, Texas 76203-5017, United States. *^c^*Department of Physical Sciences and Mathematics, Wayne State College, 1111 Main Street, Wayne, Nebraska 68787, USA. *^d^*Department of Chemistry, Brock University, 1812 Sir Isaac Brock Way, St. Catharines, Ontario, Canada L2S 3A1.

**
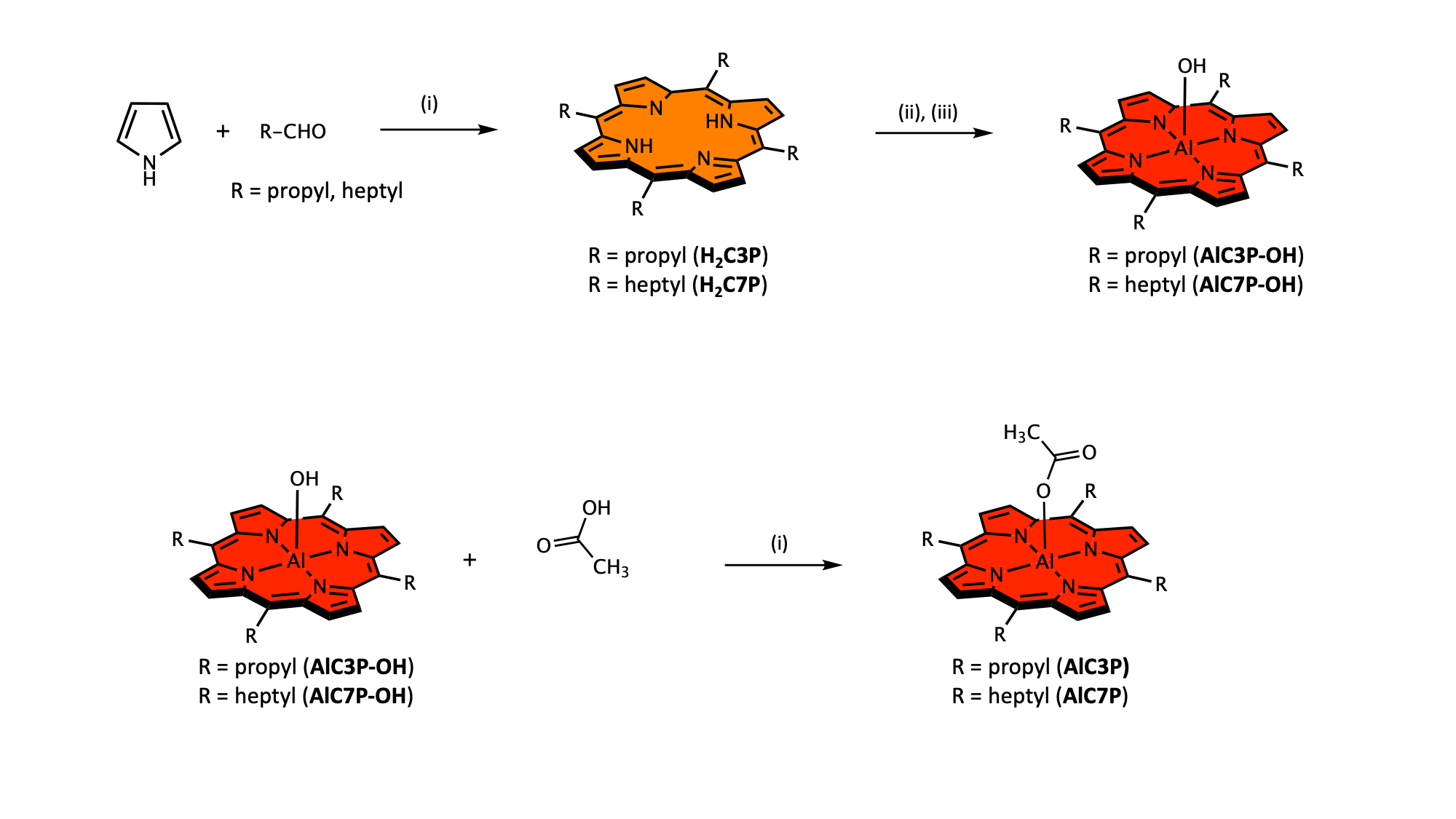
**

**Scheme S1**. Synthesis of the aluminum(III) porphyrin precursors. *Reaction conditions*: (i) BF_3_.OEt_2_, CH_2_Cl_2_, stirring under N_2_ for 3 h.; *p*-chloranil, 12 h. (ii) AlMe_3_, Toluene, (iii) H_2_O.


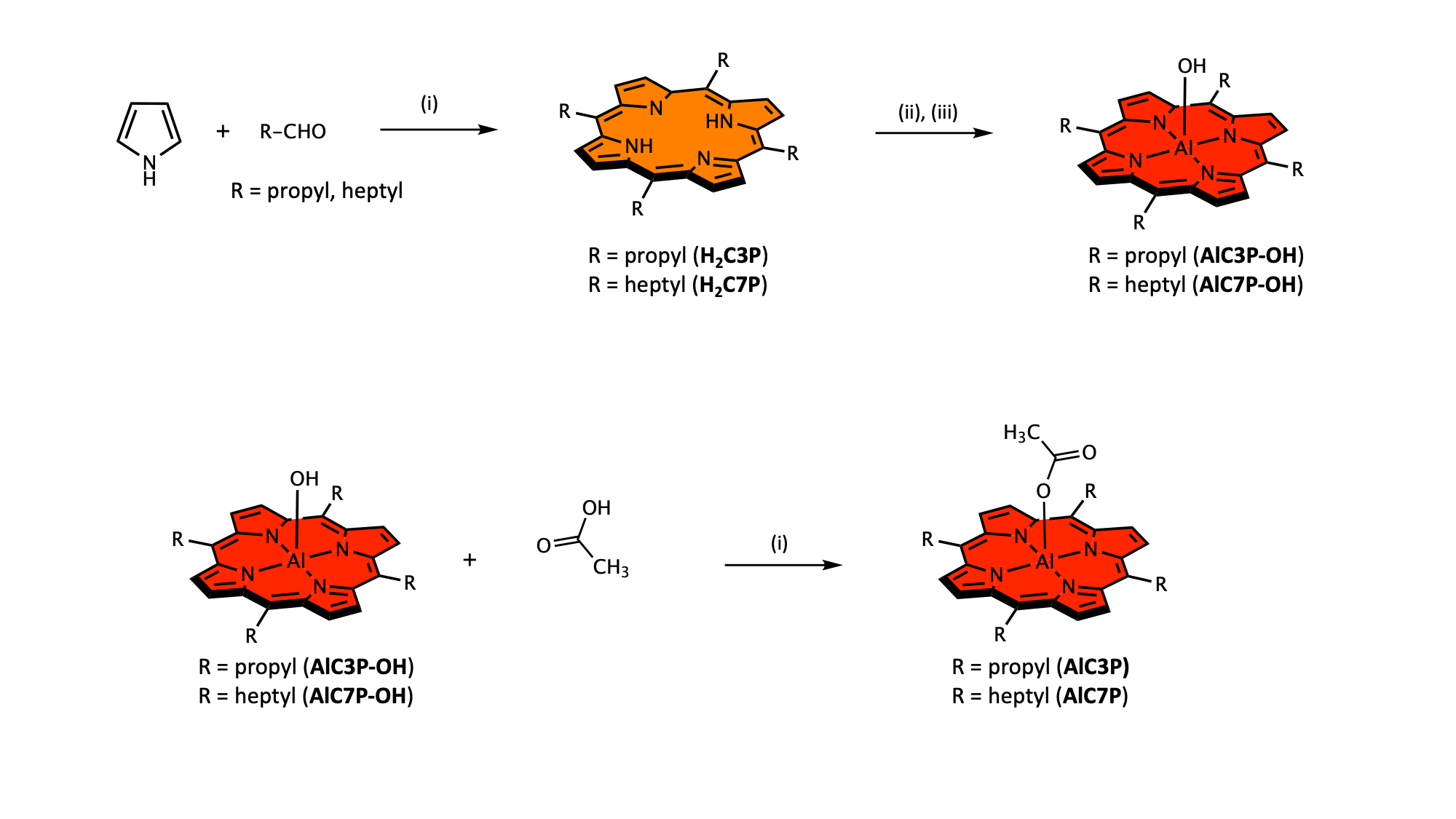


**Scheme S2**. Synthesis of the reference aluminum(III) porphyrins. *Reaction conditions*: (i) CH_2_Cl_2_, stirring under N_2_.

**
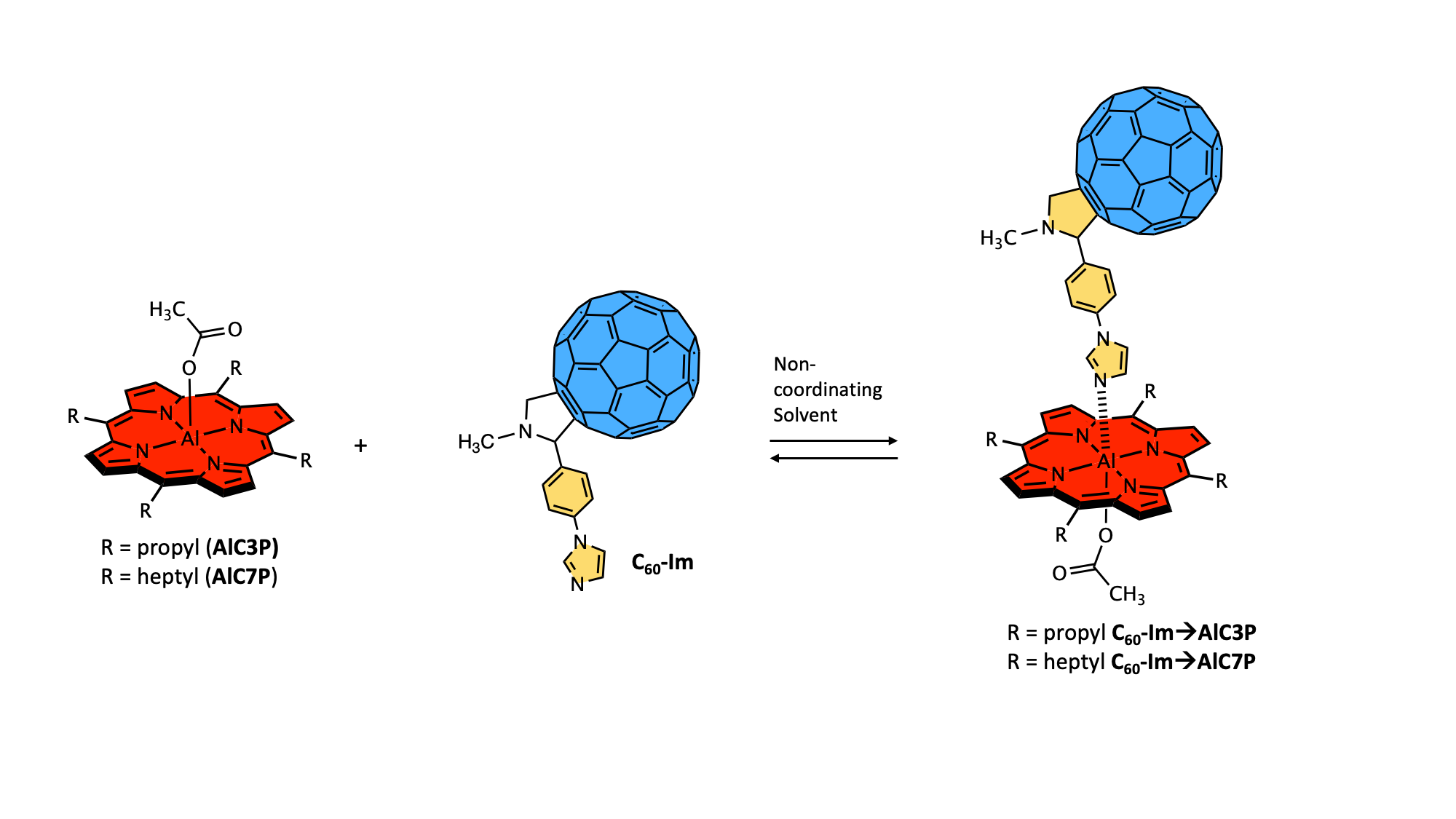
**

**Scheme S3**. Self-assembly of dyads C_60_-Im🡪AlC3P and C_60_-Im🡪AlC7P.

***Synthesis of 5,10,15,20-tetrapropylporphyrin (H_2_C3P)***. Pyrrole (1.84 g, 1.9 mL, 27.4 mmol) and butyraldehyde (2.0 g, 2.5 mL, 27.7 mmol) were dissolved in dry CH_2_Cl_2_ (500 mL). The flask was flushed with N_2_ for 1 h, followed by the addition of BF_3_.Et_2_O (0.04mL) and the solution was stirred at room temperature in the dark for 3 h. *p*-Chloranil (6.80 g, 27.7 mmol) was added to the red solution under a stream of N_2_ and the reaction mixture was then further stirred overnight for 12 h at room temperature. The dark solution was filtered with celite and concentrated under reduced pressure and loaded on a Al_2_O_3_ column. The column was eluted with CH_2_Cl_2_ to obtain the desired product. Yield: 157 mg (4.8%). ESI MS: *m/z* 479.2915 for [M+H]^+^, calculated 479.3170 for C_32_H_39_N_4_^+^. ^1^H NMR (400 MHz, CDCl_3_): *δ, ppm* 9.48 (s, 8H), 4.93 (t, 8H, *J* = 7.94 Hz), 2.55 (sextet, 8H, *J* = 7.58 Hz), 1.34 (t, 12H, *J* = 7.34 Hz), –2.65 (s, 2H).

***Synthesis of 5,10,15,20-tetrapropylaluminum(III) porphyrin (AlC3P-OH)*.** H_2_C3P (51.5 mg, 0.108 mmol) was placed in a flask in 5.5 mL of dry toluene was added trimethylaluminum (0.2 mL, 2.0 M in hexane) under an atmosphere of N_2_ in the dark. The solution turned from purple to green. The resulting solution was stirred at room temperature overnight for 18h, at which time 0.2 mL of water was added dropwise and stirring was continued for a further 21 hrs. The toluene was removed, and the residue was purified by alumina column chromatography using CH_2_Cl_2_, then CH_2_Cl_2_:CH_3_OH (= 98:2) eluent to obtain a pure solid as dark green solid. Yield: 51 mg (91%). ESI MS: *m/z* 521.2742 for [M+H]^+^, calculated 521.2861 for C_32_H_38_AlN_4_O^+^; *m/z* 562.3131 for [M+H+CH_3_CN]^+^. ^1^H NMR (400 MHz, CDCl_3_+drops of CD_3_OD): *δ, ppm* 9.58 (s, 8H), 4.78 (t, 8H, *J* = 7.94 Hz), 2.58 (sextet, 8H, *J* = 7.71 Hz), 1.30 (t, 12H, *J* = 7.28 Hz).

***Synthesis of AlC3P****.* AlC3P-OH (30 mg, 0.058 mmol) and acetic acid (21 mg, 20 µL, 0.35 mmol) were dissolved in 5 mL CH_2_Cl_2_ and stirred under N_2_ for 1 h. The solution was washed with water 3 times to remove excess acetic acid and dried over anhydrous Na_2_SO_4_. The CH_2_Cl_2_ was removed to obtain a pure compound as a dark green solid. Yield: 31 mg (95%). ESI MS: *m/z* 562.3130 for [M]^+^, calculated 562.2888 for C_34_H_39_AlN_4_O_2_. ^1^H NMR (400 MHz, CDCl_3_): *δ, ppm* 9.59 (s, 8H), 4.76 (t, 8H, *J* = 7.60 Hz), 2.47 (m, 8H), 1.20 (m, 12H), –1.69 (s, 3H).

***Synthesis of AlC7P****.* AlC7P-OH (30 mg, 0.04 mmol) and acetic acid (16 mg, 15 µL, 0.26 mmol) were dissolved in 5 mL CH_2_Cl_2_ and stirred under N_2_ for 1 h. The solution was washed with water 3 times to remove excess acetic acid and dried over anhydrous Na_2_SO_4_. The CH_2_Cl_2_ was removed to obtain a pure compound as a dark green solid. Yield: 30 mg (95%). ESI MS: *m/z* 787.5686 for [M+H]^+^, calculated 787.5465 for C_50_H_72_AlN_4_O_2_^+^; 768.5525 for [M–OAc+CH_3_CN]^+^; ^1^H NMR (400 MHz, CDCl_3_): *δ, ppm* 9.58 (s, 8H), 4.77 (t, 8H, *J* = 7.98 Hz), 2.46 (quintet, 8H, *J* = 7.68 Hz), 1.67 (quintet, 8H, *J* = 7.50 Hz), 1.49 (quintet, 8H, *J* = 7.23 Hz) 1.32 (m, 16H), 0.89 (t, 12H, *J* = 6.72 Hz), –1.65 (s, 3H).

***Synthesis of (AlC3P)_2_****.* AlC3P-OH (25 mg, 0.048 mmol) and oxalic acid (2.14 mg, 0.0238 mmol) were dissolved in 3 ml dry CH_2_Cl_2_ and stirred under N_2_ for 1 h. The CH_2_Cl_2_ was removed to obtain a pure compound as a dark green solid. Yield: 27 mg (100%). ^1^H NMR (400 MHz, CDCl_3_): *δ, ppm* 9.59 (s, 8H), 9.06 (s, 8H), 4.78 (bm, 8H), 4.48 (bm, 8H), 2.42 (bm, 8H), 2.15 (bm, 8H), 1.26 (bm, 24H).

***Synthesis of (AlC7P)_2_****.* AlC7P-OH (20.5 mg, 0.0276 mmol) and oxalic acid (1.24 mg, 0.0138 mmol) were dissolved in 3 ml dry CH_2_Cl_2_ and stirred under N_2_ for 30 min. The CH_2_Cl_2_ was removed to obtain a pure compound as a dark green solid. Yield: 22 mg (100%). ^1^H NMR (400 MHz, CDCl_3_): *δ, ppm* 9.60 (s, 8H), 9.07 (s, 8H), 4.80 (bm, 8H), 4.48 (bm, 8H), 2.43 (bm, 8H), 2.17 (bm, 8H), 1.84 (bm, 8H), 1.67, (bm, 8H), 1.48 (bm, 16H), 1.32 (bm, 16H), 1.02 (bm, 16H), 0.89 (bm, 24H).

**Physical Methods**

***NMR and mass spectroscopy*.** NMR spectra were recorded with a Bruker 400 MHz NMR spectrometer using CDCl_3_ as the solvent. ESI mass spectra were recorded on a Bruker MicroTOF-III mass spectrometer.

***Electrochemistry*.** Cyclic and differential pulse voltammetric experiments (CH_2_Cl_2_, 0.1 M tetrabutylammonium hexafluorophosphate, (TBA.PF_6_)) were performed on a BAS Epsilon electrochemical analyzer (working electrode: Pt, auxiliary electrodes: Pt wire, reference electrode: Ag wire). The ferrocene couple (*E*_1/2_ (Fc^+^/Fc) = 0.46 V in CH_2_Cl_2_, 0.1 M TBA.PF_6_ under our experimental conditions)^[1]^ was used to calibrate the redox potentials.

***Optical spectroscopy*.** Steady-state UV-visible absorption spectra were recorded with a Cary 100 UV-VIS spectrometer. The concentrations of the samples used for these measurements ranged from 10^−6^ M (porphyrin Soret band) to 10^−5^ M (Q-bands) solutions. Steady-state fluorescence spectra were recorded using a Photon Technologies International Quanta Master 8075-11 spectrofluorometer, equipped with a 75 W xenon lamp, running FelixGX software. An excitation wavelength of 550 nm was used, and the optical density (OD) was held constant at 0.2 for all the compounds. The fluorescence lifetimes were evaluated using a Horiba Yvon Nanolog Spectrofluorometer equipped with time-correlated single-photon counting and nanoLED excitation sources. A right-angle detection method was used.

***DFT Computational studies.*** The structures studied herein were assembled on a local pc using the GaussView 6.0 program. During the assembly process, the bond lengths, bond angles, and dihedrals were carefully chosen based on valence bond and hybridization theory. The GaussView “clean” function was avoided as it is known to distort the porphyrin structure. Once the structures were assembled, they were uploaded to a supercomputer where final editing for optimization was completed. The self-consistent field (SCF) convergence constraints and the DFT grid utilized in the calculations were the *Gaussian 16* default values, Tight and UltraFine, respectively. The optimization of each chemical species continued until the maximum force, root mean square (rms) force, maximum displacement, and rms displacement reached the default *Gaussian 16* minima, and the predicted energy change upon another geometry optimization cycle was in the range of -5x10^-9^ A.U. To avoid SCF (Self Consistent Field) convergence failure, initial DFT computations of the structures herein were performed on a supercomputer via the *Gaussian 16* software suite *sans* symmetry constraints *in-vaccuo* utilizing the modest B3LYP/3-21G* split-valence model chemistry. After convergence to a stationary point on the Born-Oppenheimer surface with the B3LYP/3-21G* model chemistry, the resulting geometry was then optimized using the B3LYP/6-31G model chemistry under the same constraints. Next, polarization functions were added to the B3LYP/6-31G optimized structures forming the B3LYP/6-311G(d,p) model chemistry and the structures were again optimized to a stationary point on the Born-Oppenheimer surface. Upon final optimization, a single point energy calculation was performed using the Cam-B3LYP/6-311G(d,p) model chemistry. The FMO (HOMO-2, HOMO-1, HOMO, LUMO, LUMO+1, and LUMO+2) cube files were generated on the supercomputer using the G16 cubegen function. (cubegen 0 MO=*nnn* *filename*.fch *filename*-HOMO.cub). Where “*nnn”* is the MO number of the HOMO and “*filename”* is the name of the formatted check file. At the same time cube files of the electron density and potential of the ground state were generated also using the G16 cubegen function. (cubegen 0 density=scf *filename*.fch *filename* -Den.cub 0 h) and (cubegen 0 potential=scf *filename*.fch *filename* -POT.cub 0 h).

***Femtosecond and nanosecond laser flash photolysis***. Femtosecond transient studies were performed using an Ultrafast Femtosecond Laser Source (Astrella) by Coherent, which incorporates a diode, mode-locked Ti: Sapphire laser (Vitara), and diode-pumped intracavity doubled Nd: YLF laser (Revolution) to generate a fundamental compressed laser of 800 nm and power output of 5.24 W. A Helios transient absorption spectrometer coupled with a femtosecond harmonics generator, both provided by Ultrafast Systems LLC, will be used for optical detection. The source for the pump pulse is derived from the fundamental output of Astrella (compressed output 5.24 W, pulse width 100 fs, 800 nm at a repetition rate of 1 kHz) by introducing 95% of the beam into the OPA while the other 5% is sent to the delay line and white light generating crystal. The beam sent through the OPA is termed the pump beam, as it is used to excite the sample. The beam sent through the delay line and crystal is termed the probe beam as it shows what spectral changes occur in the sample with time. The OPA takes the 800 nm fundamental and converts it into a specific wavelength in the 400 – 2200 nm range, which allows the excitation wavelength to be selected. Kinetic traces at appropriate wavelengths were assembled from the time-resolved spectral data. Data analysis was performed using Surface Xplorer software. All measurements were conducted in degassed solutions at 298 K.

***Transient EPR spectroscopy.*** Transient EPR experiments were performed using a modified Bruker EPR 200D-SRC X-band spectrometer equipped a CF935 cryostat. Time/field datasets were recorded in direct-detection mode without field modulation using diode detection, amplified using a broadband preamplifier and digitized using a LeCroy Waverunner digital oscilloscope. EPR samples were prepared by dissolving the porphyrin complex in toluene, *o*-dichlorobenzene (*o*-DCB) or the liquid crystal 4-cyano-4'-pentylbiphenyl (5CB) to a concentration of ~7 × 10^−4^ M. For the room temperature experiments on the *o*-DCB samples, the solution was placed in a flat cell and sealed with parafilm to prevent exposure to oxygen. The 5CB and toluene samples were placed in a 4-mm o.d. EPR tube and degassed by several freeze pump thaw cycles prior to the measurements. The samples were excited at 532 nm using 10 ns pulses from a Surelite Nd:YAG laser at a repetition rate of 10 Hz.


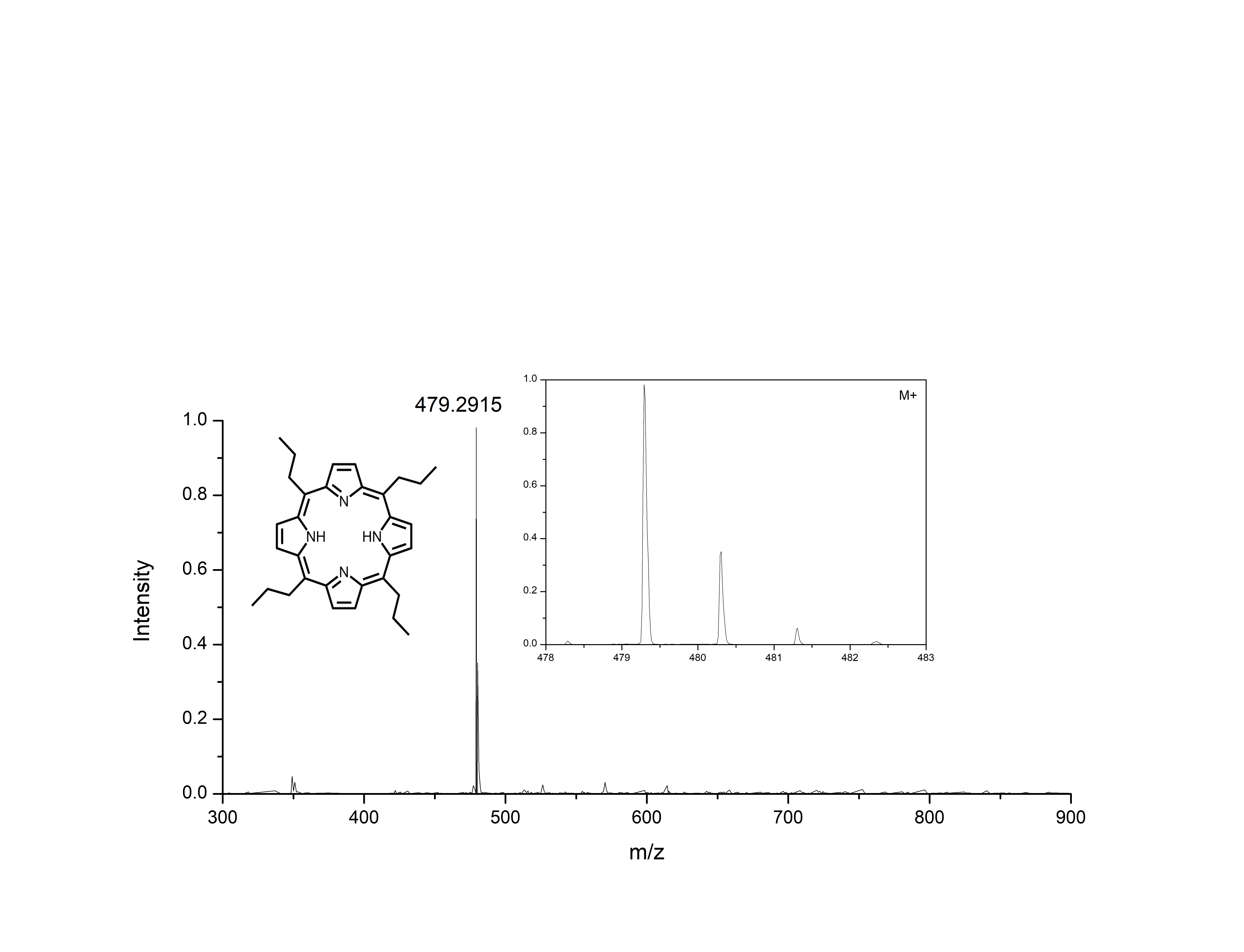


**
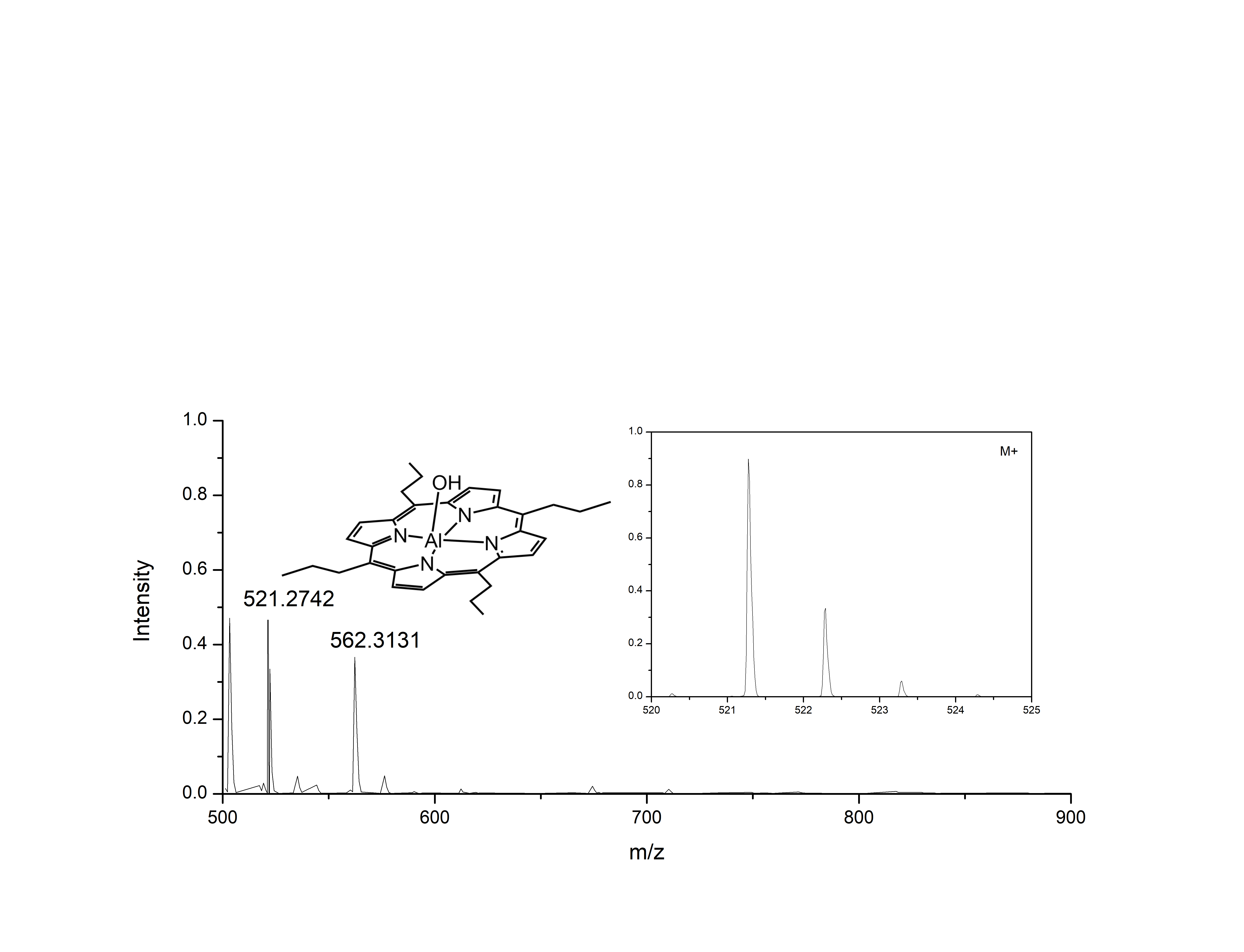
**

**Figure S1**. ESI-MS spectra of H_2_C3P and AlC3P-OH.

**
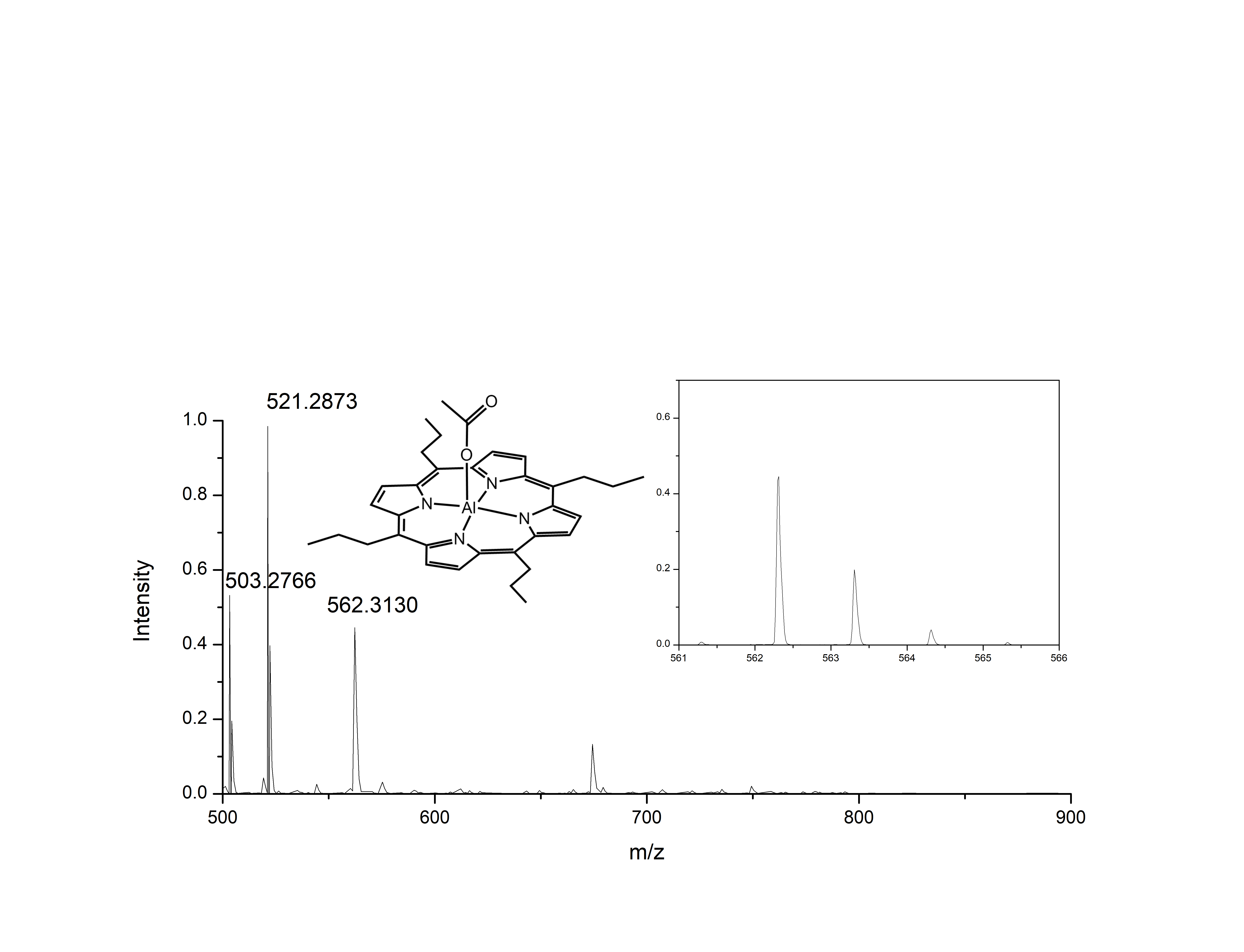
**

**
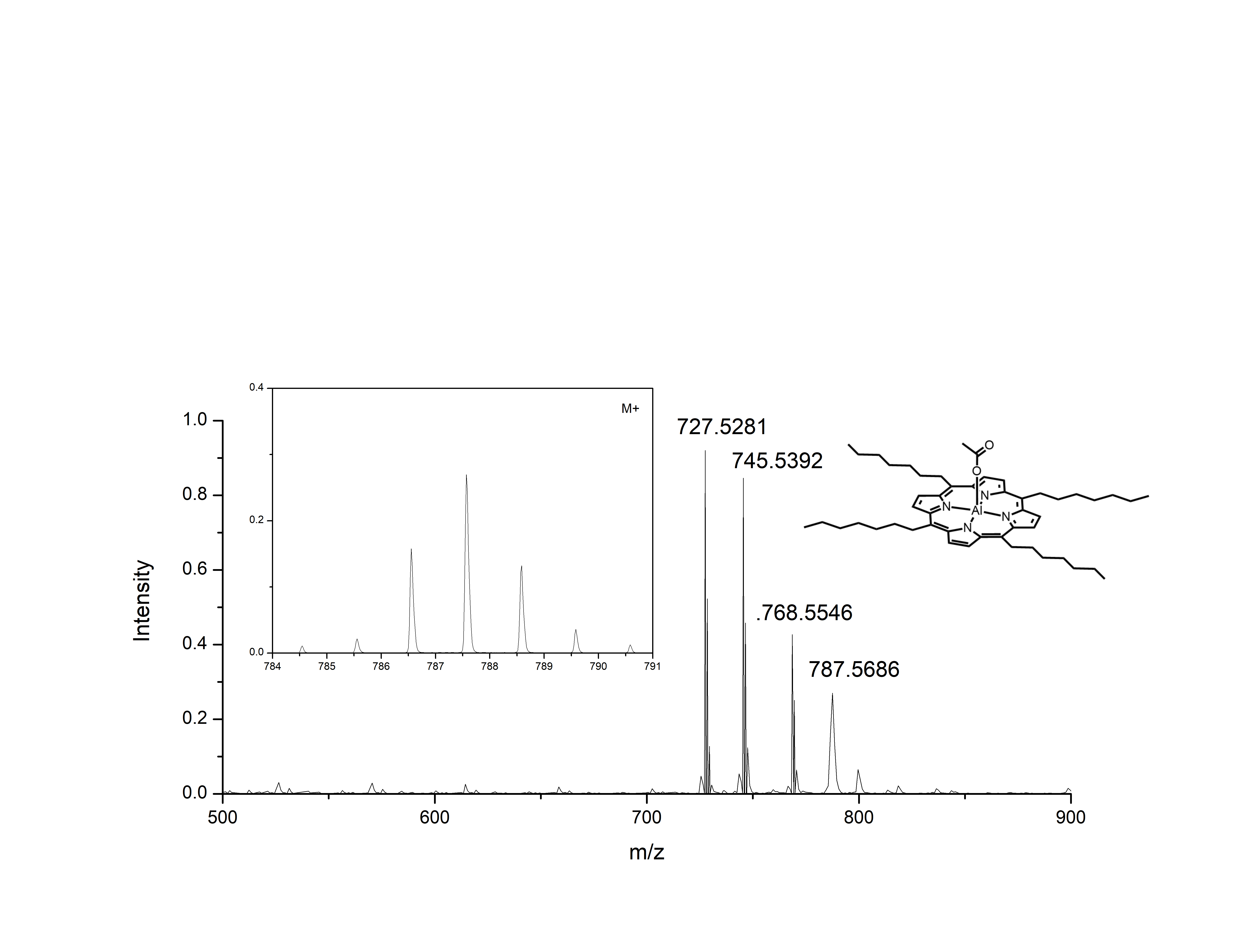
**

**Figure S2**. ESI-MS spectra of AlC3P and AlC7P.


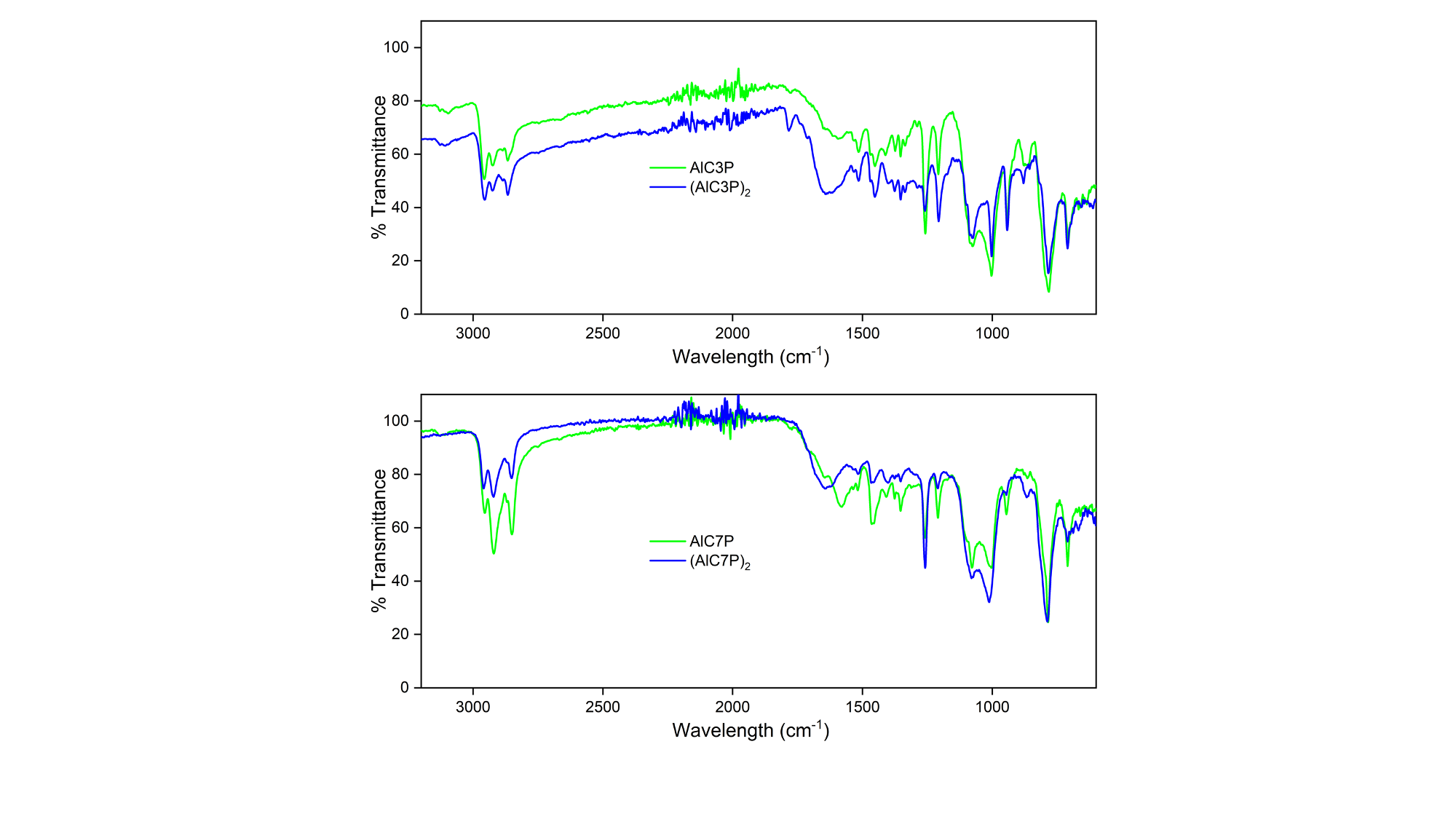


**Figure S3**. FT-IR spectra of investigated aluminum(III) porphyrin monomer and dimer systems.


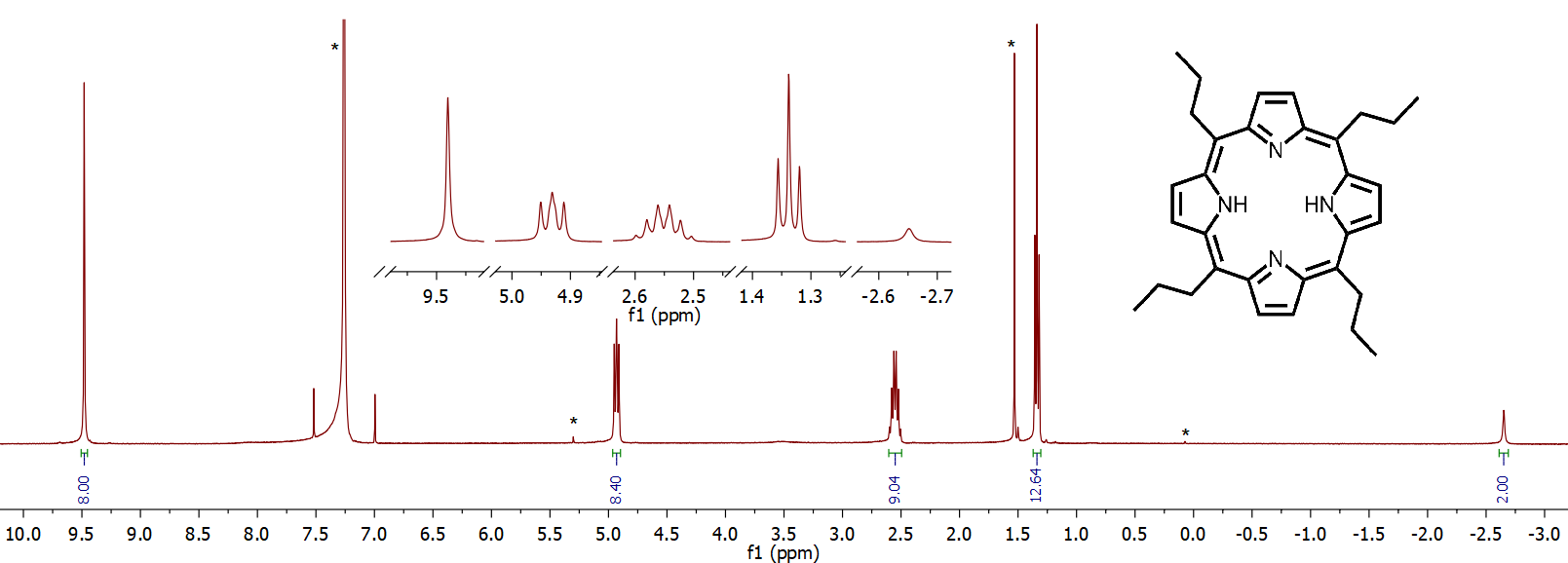


**
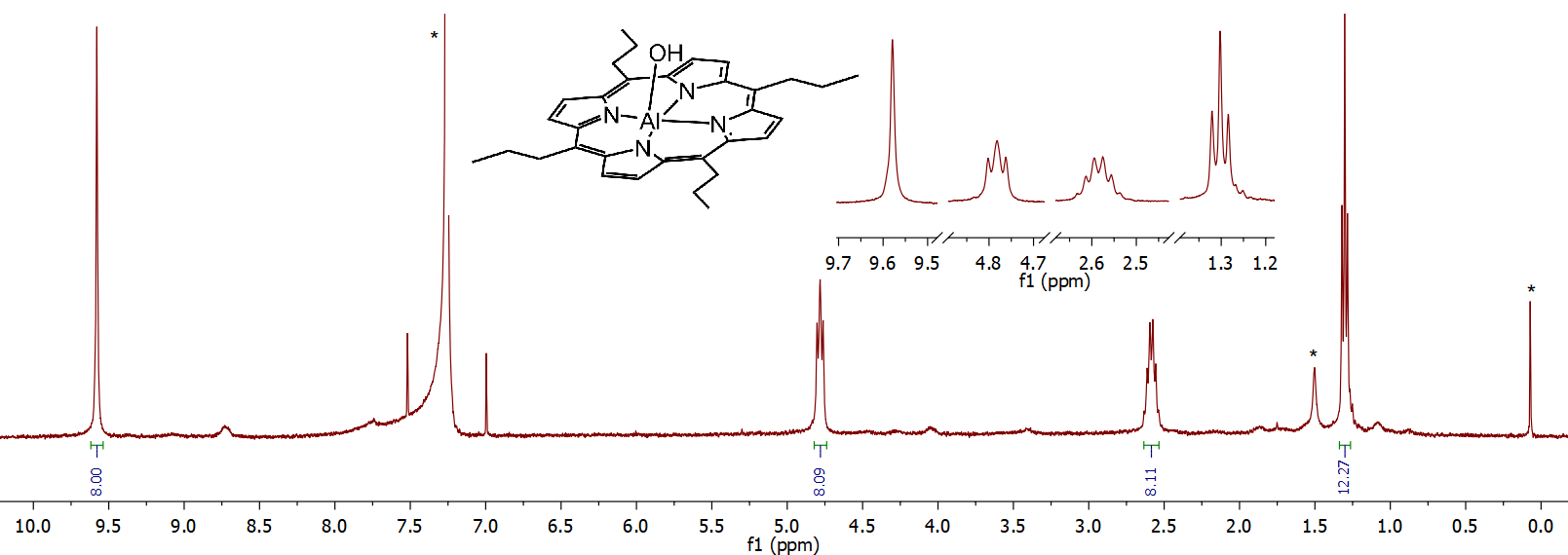
**

**Figure S4**. ^1^H NMR (400 MHz) of H_2_C3P and AlC3P-OH in CDCl_3_.


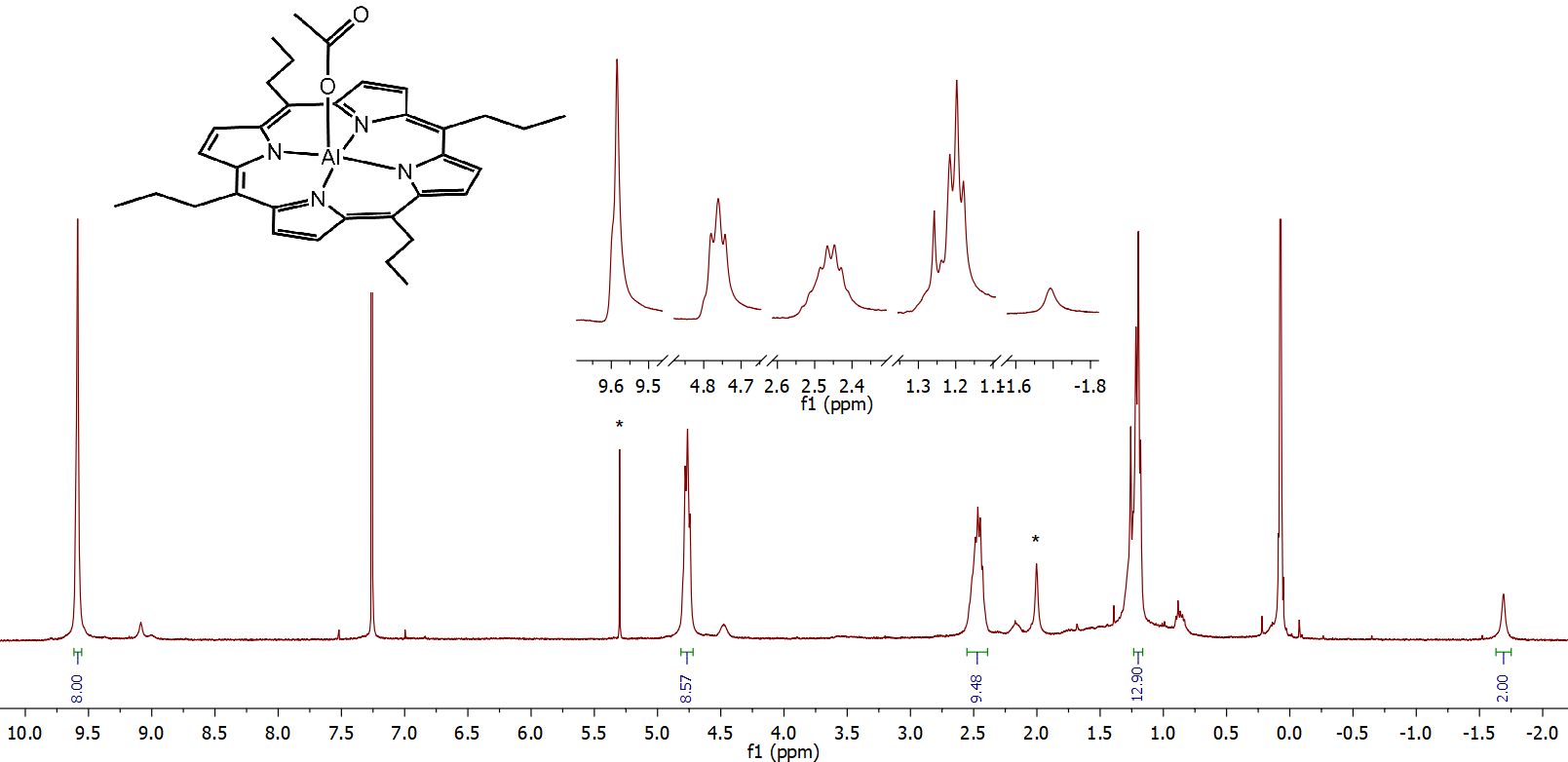


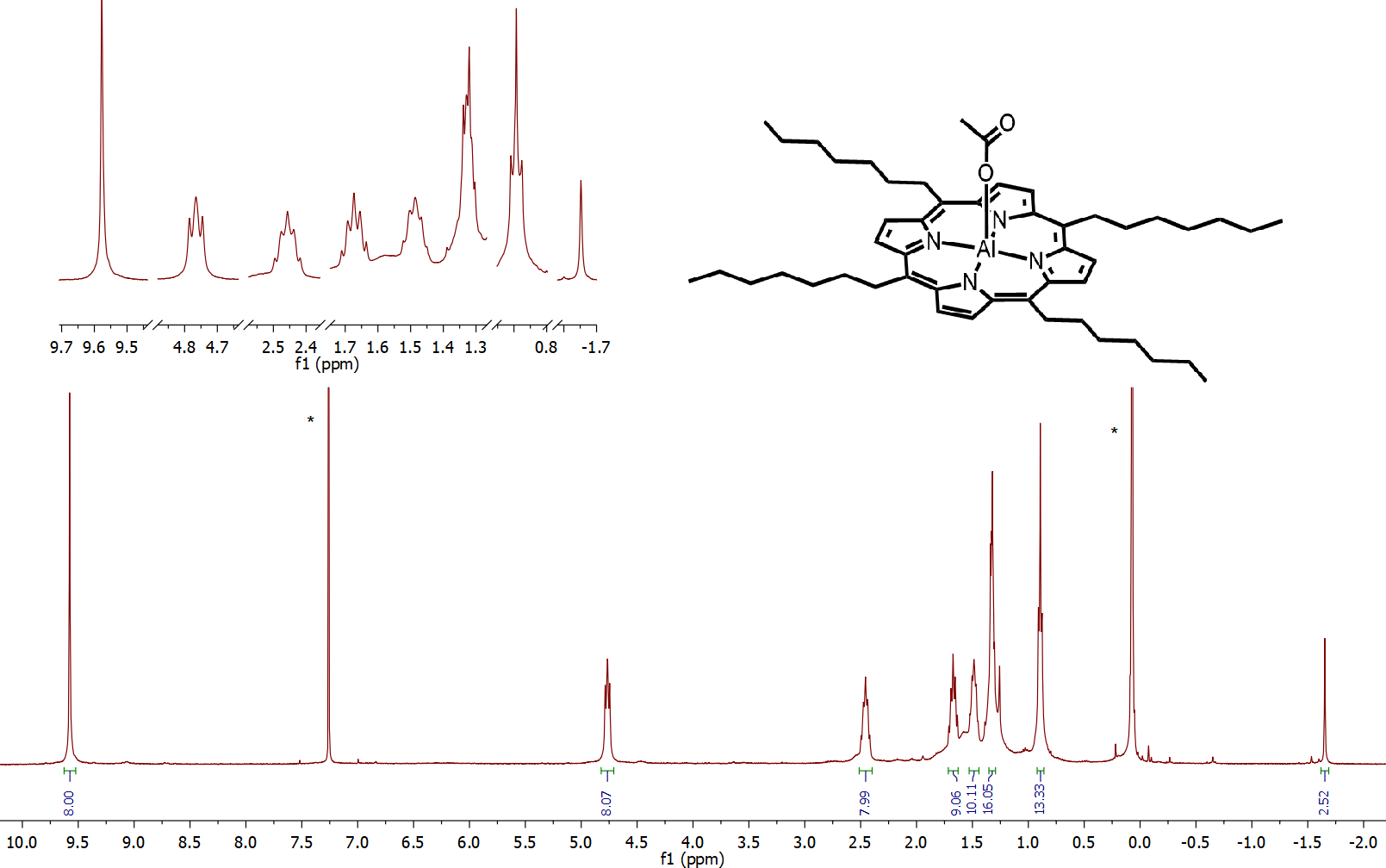


**Figure S5**. ^1^H NMR (400 MHz) spectra of AlC3P and AlC7P in CDCl_3_.


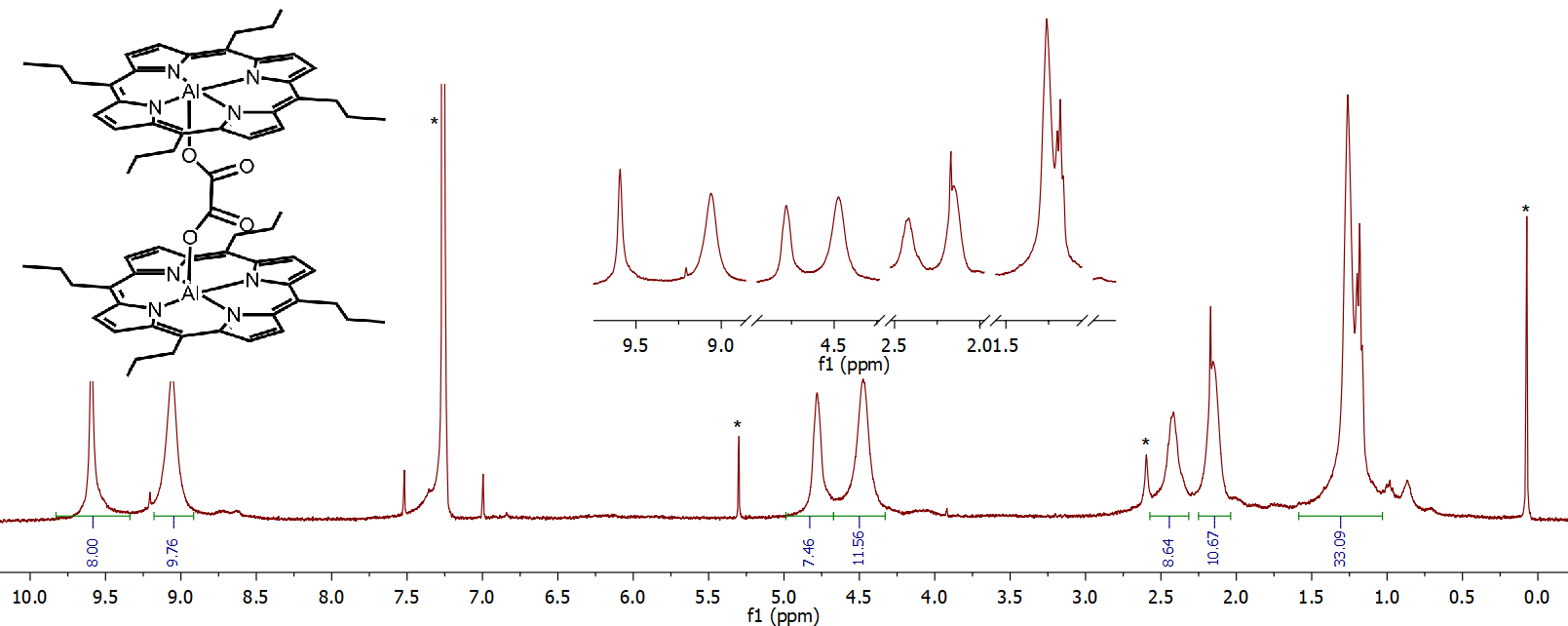

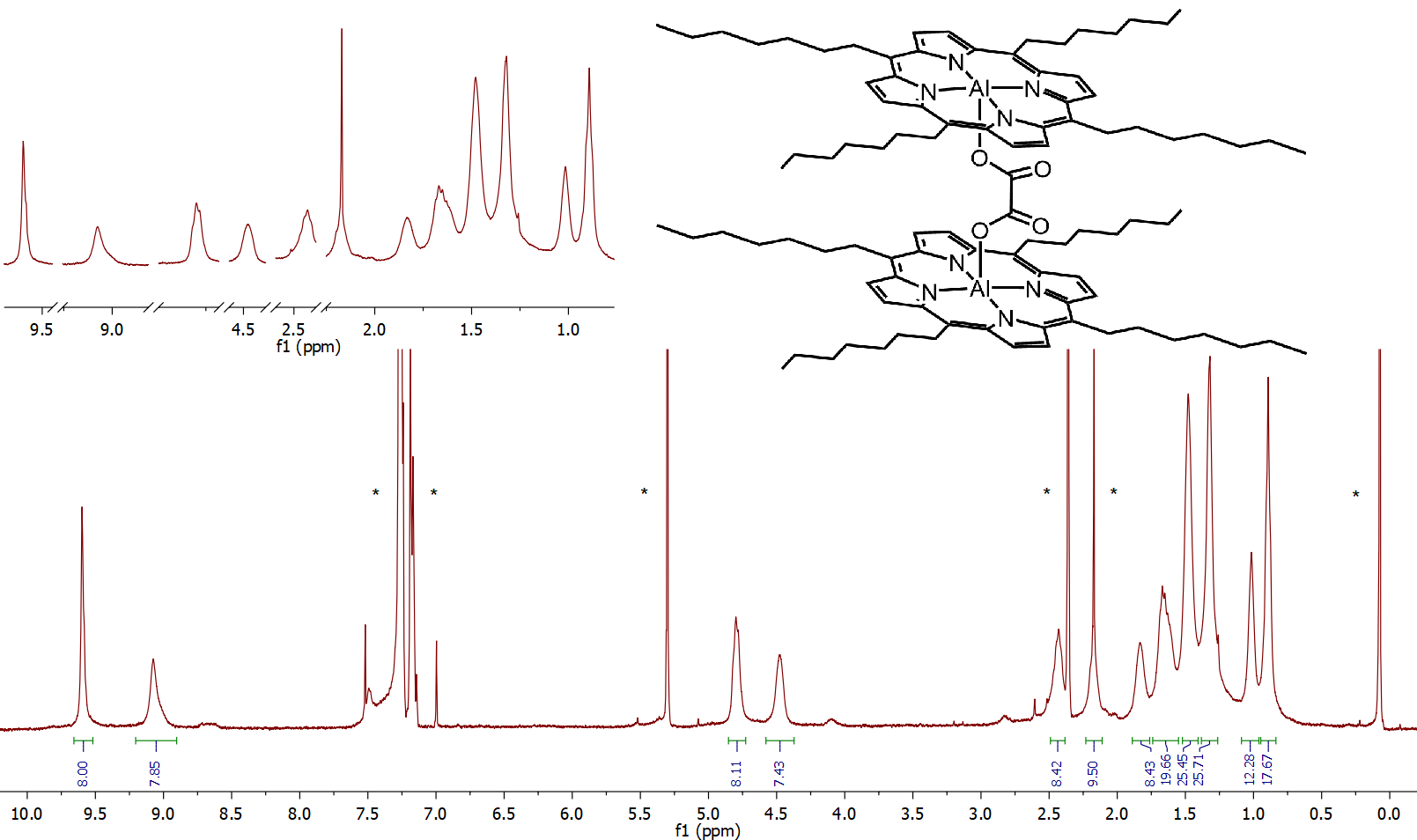


**Figure S6**. ^1^H NMR (400 MHz) of (AlC3P)_2_ and (AlC7P)_2_ in CDCl_3_.


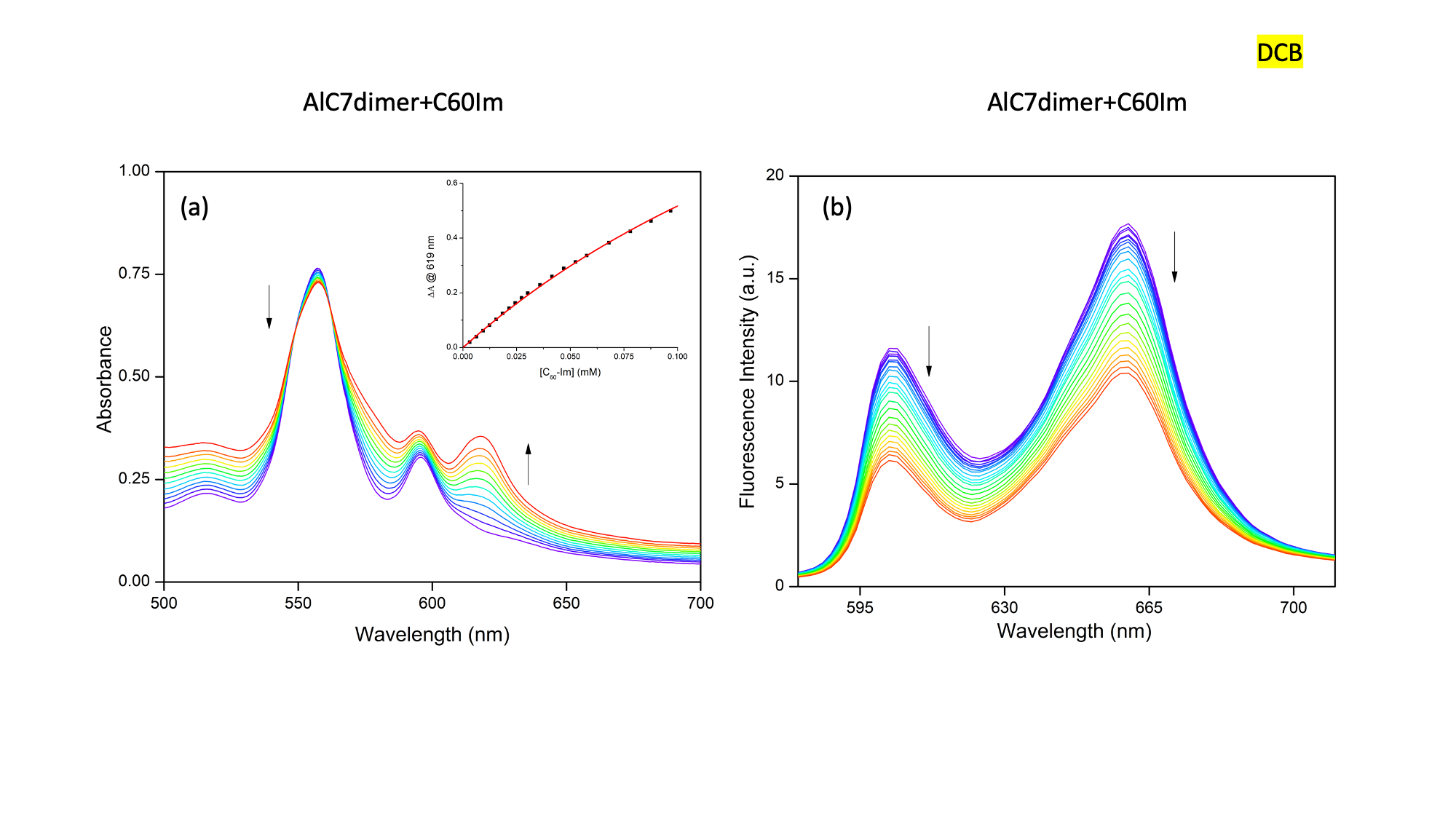


**Figure S7**. Absorption (a) and fluorescence (b) titrations of (AlC7P)_2_ *vs* C_60_-Im in *o*-DCB. C_60_-Im was added up to 4.15 × 10^−5^ M in increments from 5 – 10 μL in absorption titrations to a 1 mL (5 × 10^−5^ M) solution of (AlC7P)_2_ and up to 1.68 × 10^−4^ M in increments from 5 – 40 μL in fluorescence titrations to a 1 mL (4.27 × 10^−5^ M) solution of (AlC7P)_2_. The inset shows the Thordarson curve fit. The isosbestic point 562 nm was utilized to excite the sample.


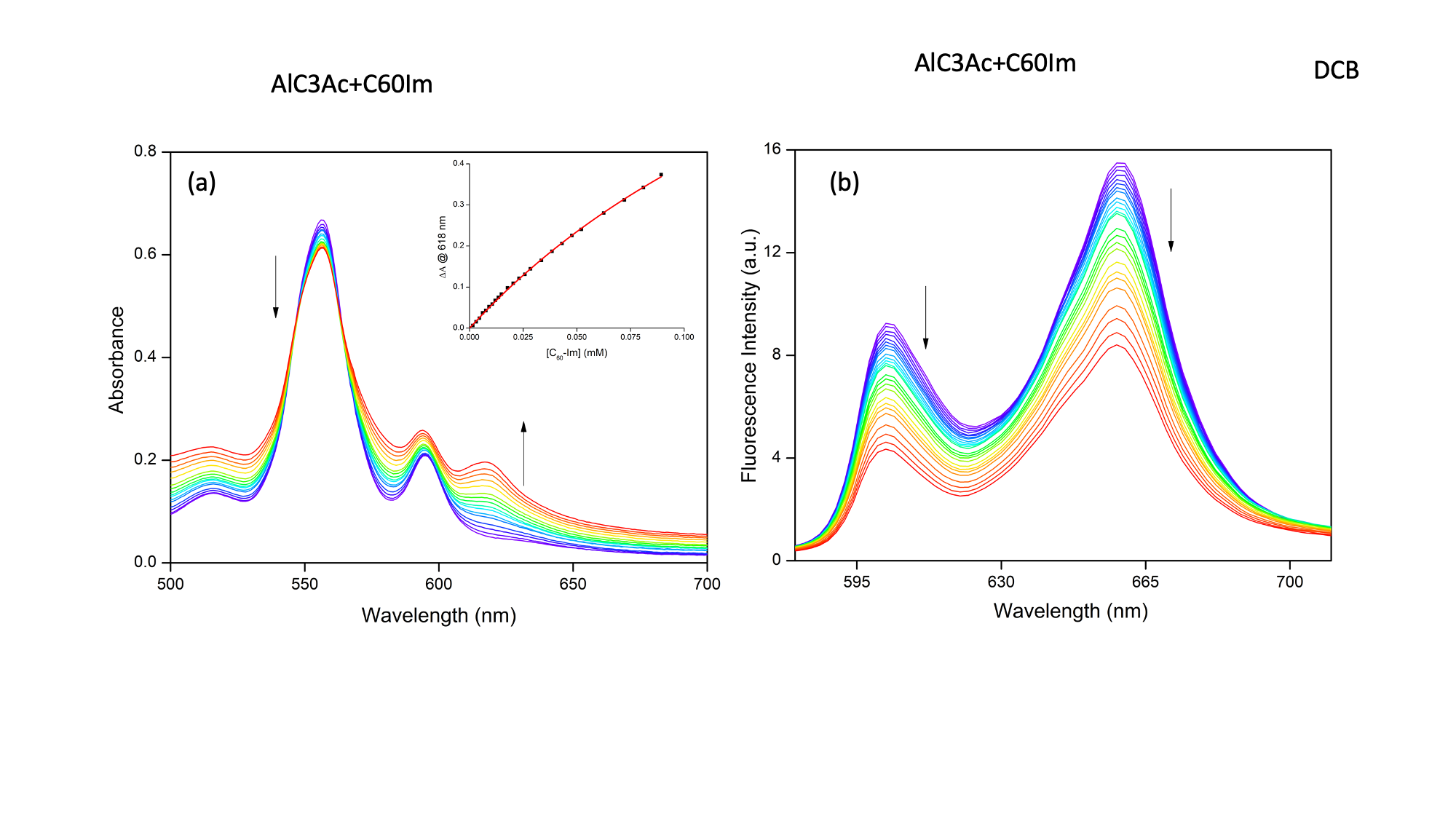


**Figure S8**. Absorption (a) and fluorescence (b) titrations of AlC3P *vs* C_60_-Im in *o*-DCB. C_60_-Im was added up to 2.84 × 10^−5^ M in increments from 5 - 10 μL in absorption titrations and up to 8.93 × 10^−5^ in increments from 5 - 50 μL in fluorescence titrations to a 1 mL (5 × 10^−5^ M) solution of AlC3P. The inset shows the Thordarson curve fit. The isosbestic point at 566 nm was utilized to excite the sample.

**
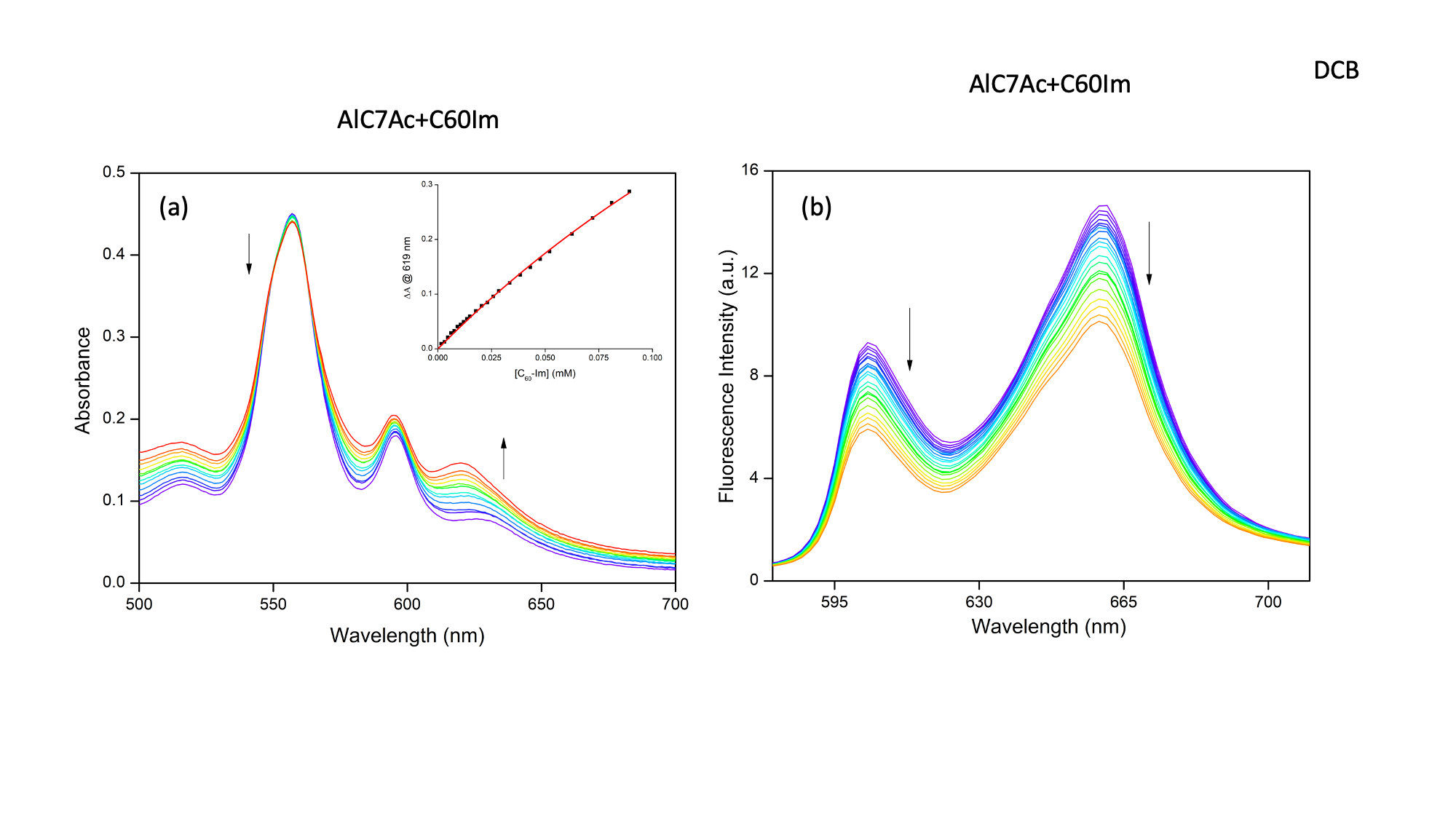
**

**Figure S9**. Absorption (a) and fluorescence (b) titrations of AlC7P *vs* C_60_-Im in *o*-DCB. C_60_-Im was added up to 1.77 × 10^−5^ M in increments from 5 - 10 μL in absorption titrations and up to 5.21 × 10^−5^ M in increments from 5 - 20 μL in fluorescence titrations to a 1 mL (5 × 10^−5^ M) solution of AlC7P. The inset shows the Thordarson curve fit. The isosbestic point at 565 nm was utilized to excite the sample.


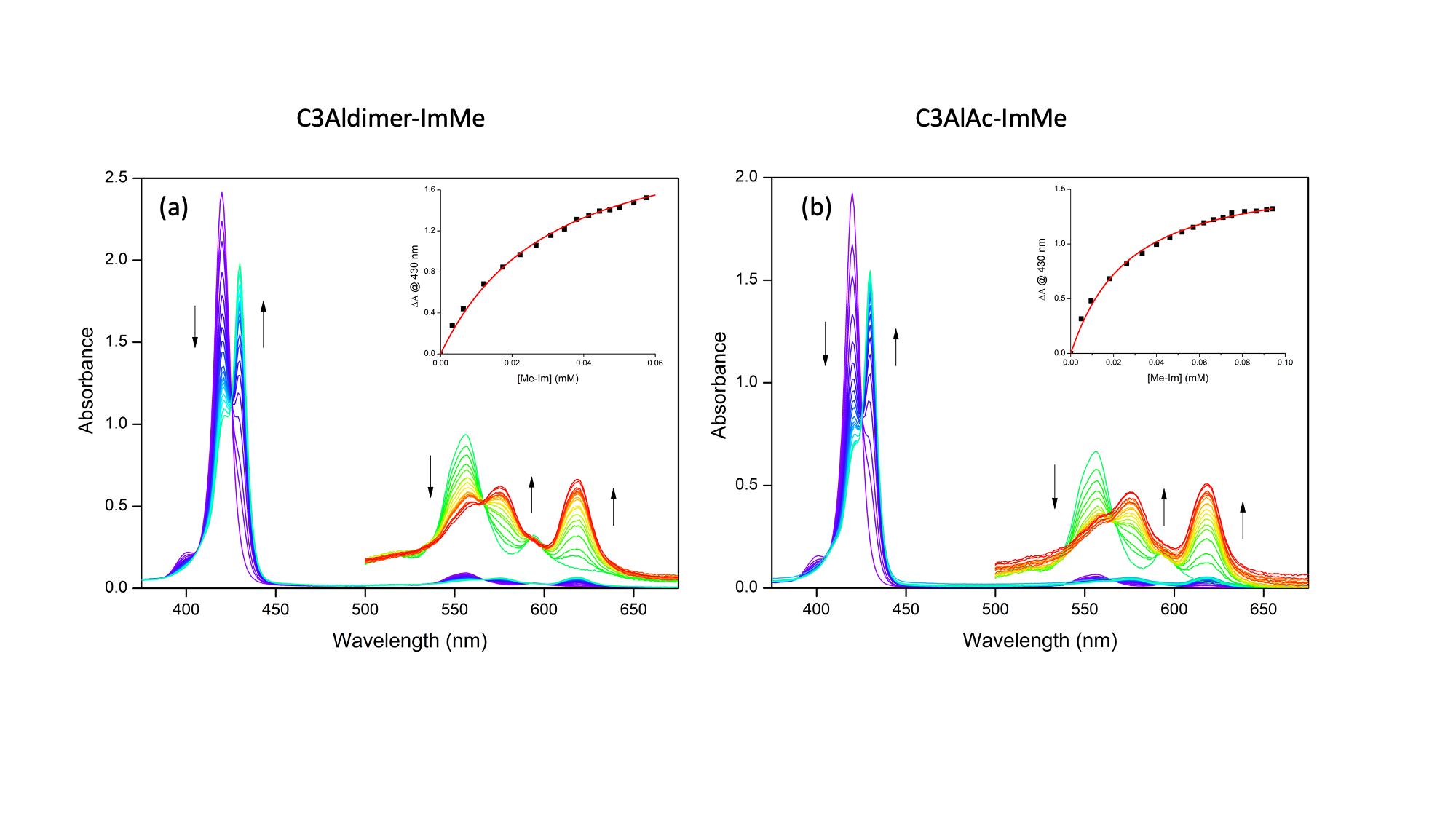


**Figure S10**. Absorption titrations of (a) (AlC3P)_2_ *vs* Me-Im: Me-Im was added up to 6.39 × 10^–5^ M in increments from 25-100 μL to a 1 mL (3.33 × 10^–6^ M) solution of (AlC3P)_2_, the inset shows the Thordarson curve fit, and (b) AlC3P *vs* Me-Im: Me-Im was added up to 9.41 × 10^–5^ M in increments from 5 – 50 μL to a 1 mL (5 × 10^–6^ M) solution of AlC3P, the inset shows the Thordarson curve fit. *o*-DCB was used as solvent.


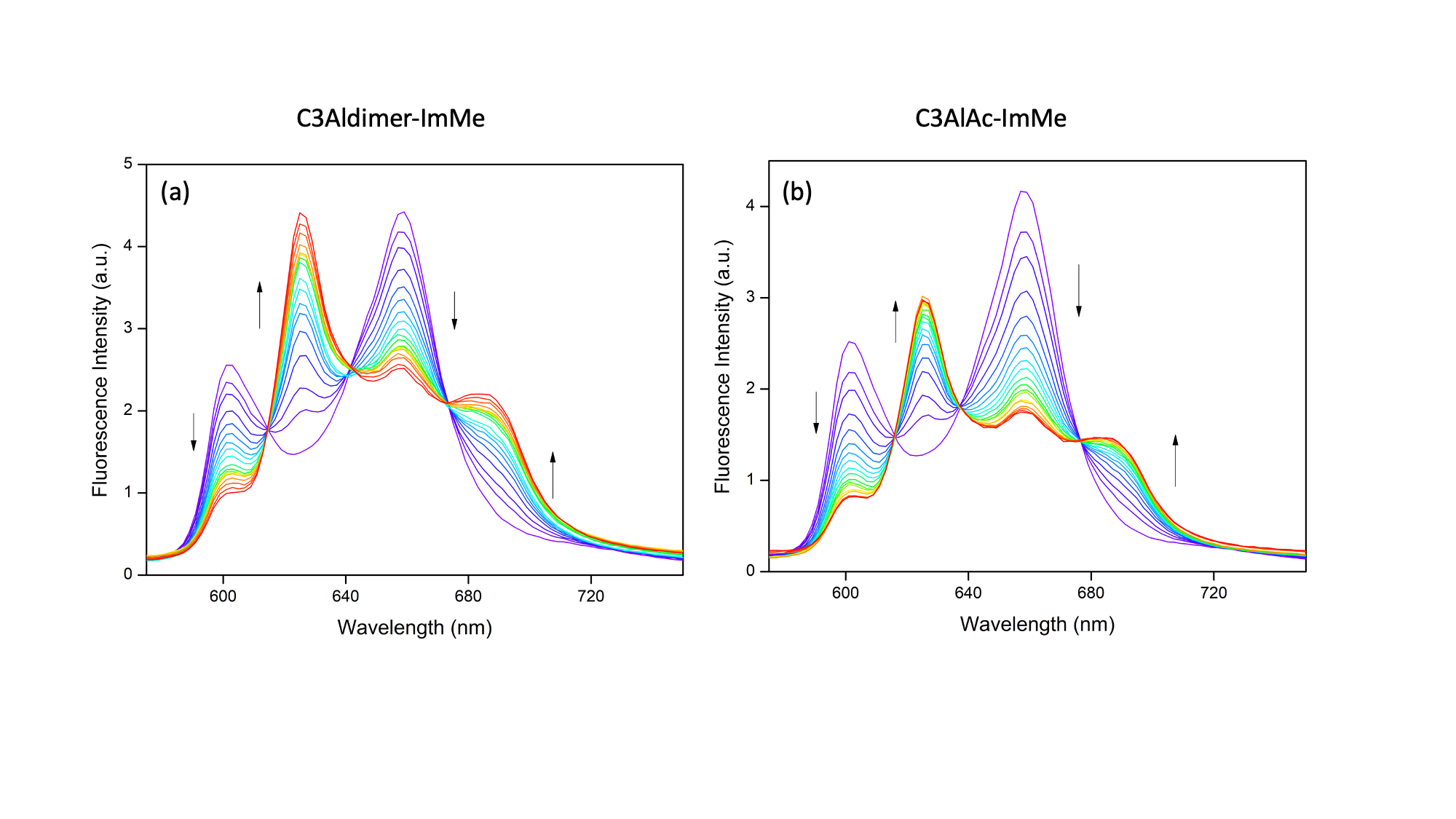


**Figure S11**. Fluorescence titrations of (a) (AlC3P)_2_ *vs* Me-Im: Me-Im was added up to 6.39 × 10^–5^ M in increments from 25-100 μL to a 1 mL (3.33 × 10^–6^ M) solution of (AlC3P)_2_, the isosbestic point was utilized to excite 565 nm the sample, and (b) AlC3P *vs* Me-Im: Me-Im was added up to 9.41 × 10^–5^ M in increments from 5 – 50 μL to a 1 mL (5 × 10^–6^ M) solution of AlC3P, the isosbestic point was utilized to excite 563 nm. *o*-DCB was used as solvent.


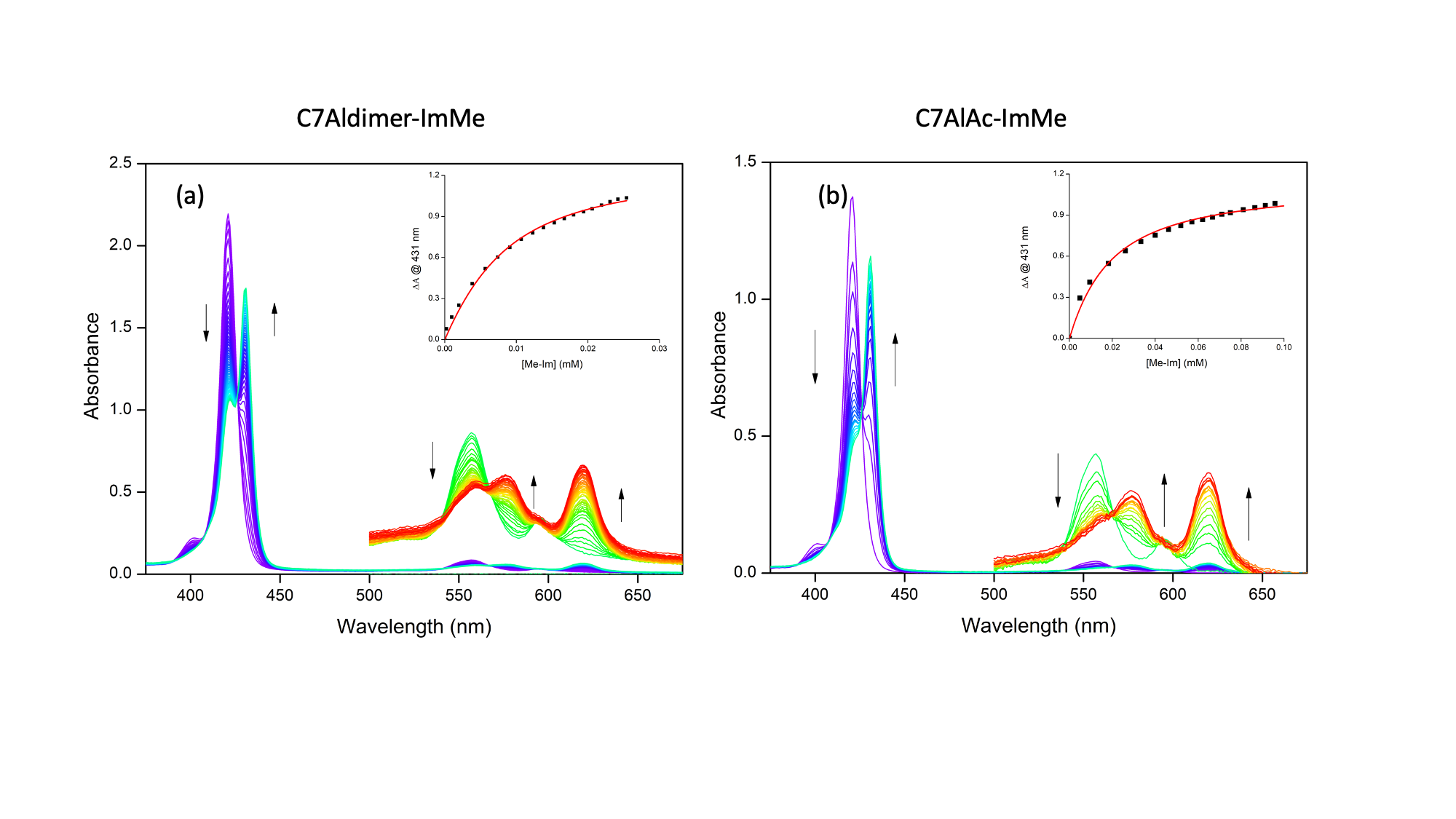


**Figure S12**. Absorption titrations of (a) (AlC7P)_2_ *vs* Me-Im: Me-Im was added up to 5 × 10^–5^ M in increments from 2.5 – 20 μL to a 1 mL (5 × 10^–6^ M) solution of (C_7_AlPor)_2_-Ox, the Thordarson curve fit, and (b) AlC7P *vs* Me-Im: Me-Im was added up to 1.04 × 10^–4^ M in increments from 25 – 100 μL to a 1 mL (5 × 10^–6^ M) solution of AlC7P, the inset shows the Thordarson curve fit. *o*-DCB was used as solvent.


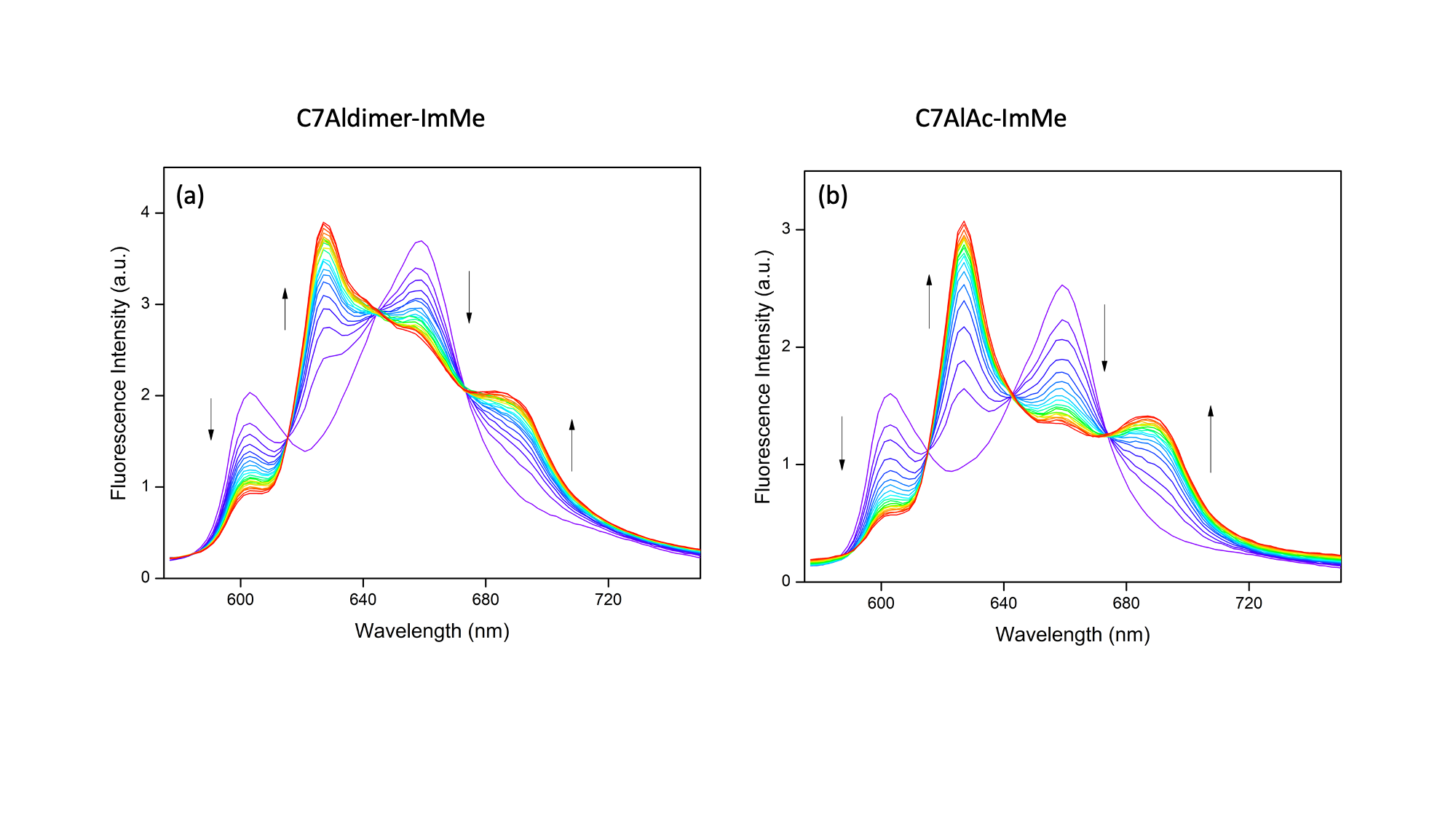


**Figure S13**. Fluorescence titration of (a) (AlC7P)_2_ *vs* Me-Im: Me-Im was added up to 5.48 × 10^–5^ M in increments of 50 μL to a 1 mL (5 × 10^–6^ M) solution of (AlC7P)_2_, the isosbestic point was utilized to excite 567 nm., and (b) AlC7P *vs* Me-Im: Me-Im was added up to 1.04 × 10^–4^ M in increments from 25 – 100 μL to a 1 mL (5 × 10^–6^ M) solution of AlC7P, the isosbestic point was utilized to excite 567 nm. *o*-DCB was used as solvent.

**Table S1**. Binding constants of self-assembled systems.

| **Titration** | **Binding Constant (M^–1^)** |
| --- | --- |
| (AlC3P)_2_ *vs* C_60_-Im | 2.30 × 10^3^ |
| (AlC7P)_2_ *vs* C_60_-Im | 3.67 × 10^3^ |
| AlC3P *vs* C_60_-Im | 4.44 × 10^3^ |
| AlC7P *vs* C_60_-Im | 2.78 × 10^3^ |
| (AlC3P)_2_ *vs* Me-Im | 3.80 × 10^4^ |
| (AlC7P)_2_ *vs* Me-Im | 7.19 × 10^4^ |
| AlC3P *vs* Me-Im | 4.31 × 10^4^ |
| AlC7P *vs* Me-Im | 5.96 ×10^4^ |


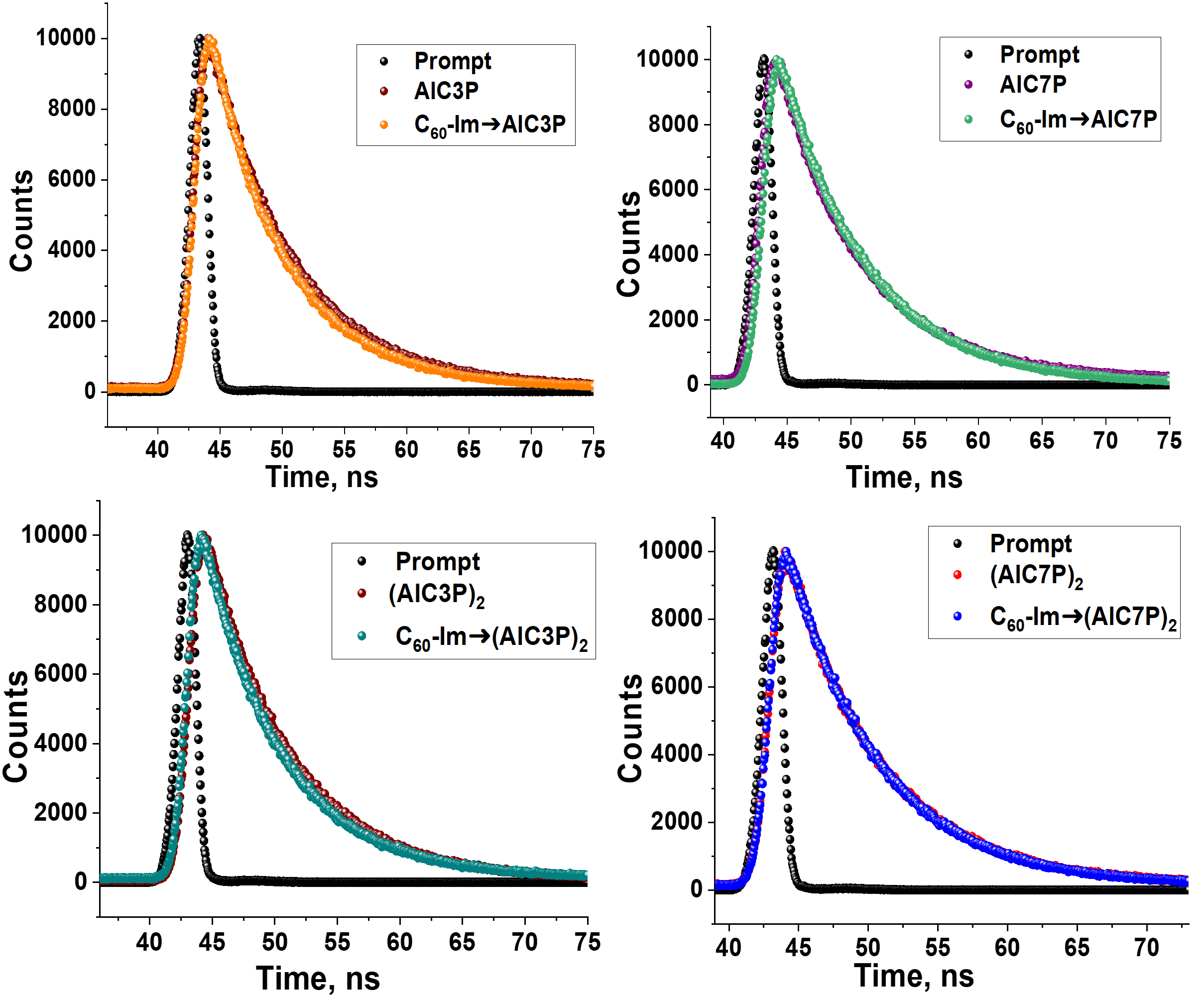


**Figure S14**. TCSPC spectra for lifetime studies of the singlet state in solvent *o-*DCB at excitation wavelength 560 nm and emission wavelength of 655 nm.


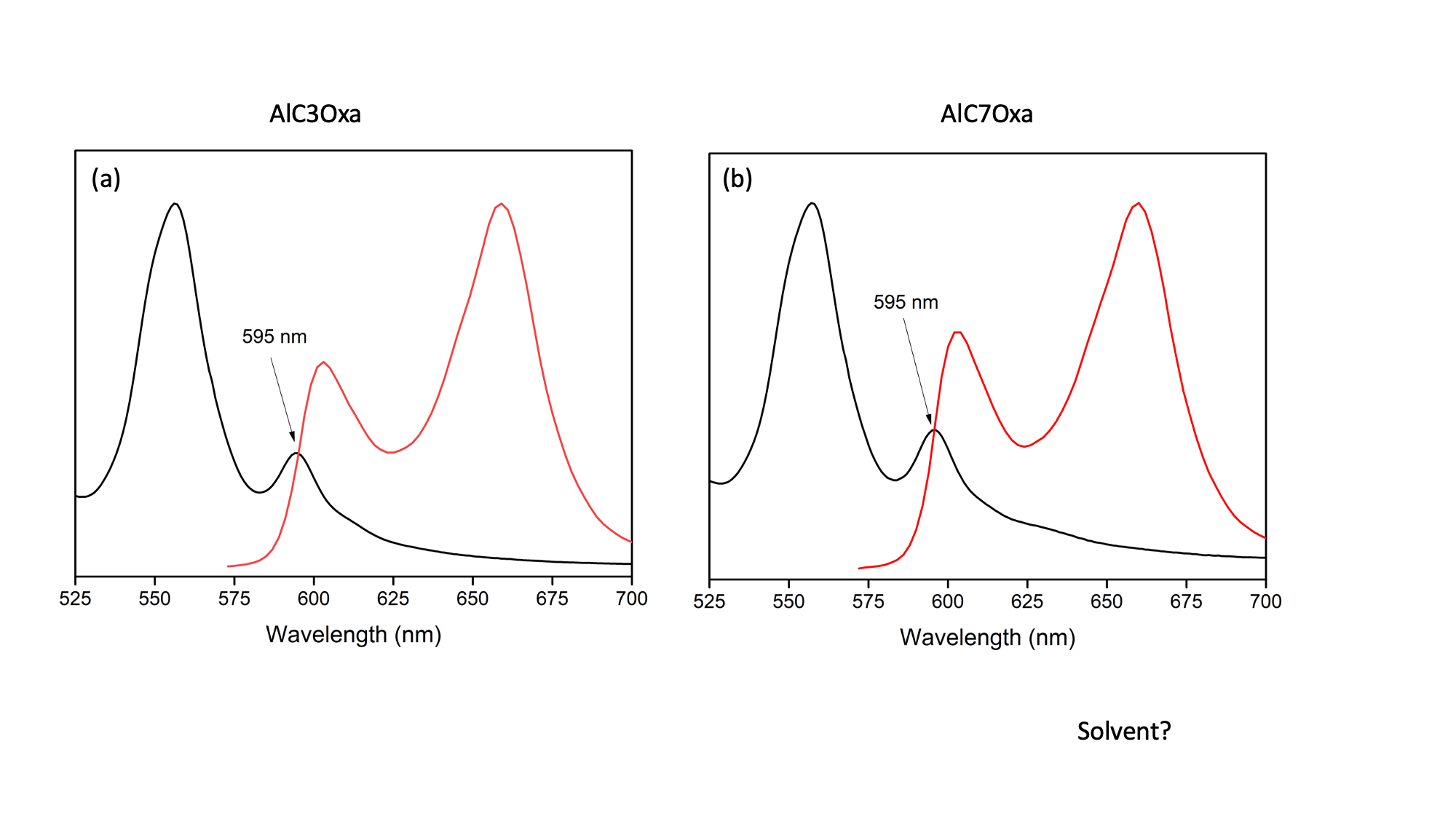


**Figure S15**. Absorption (black) and fluorescence (red) spectral overlap of (a) (AlC3P)_2_ and (b) (AlC7P)_2_ in CH_2_Cl_2_.


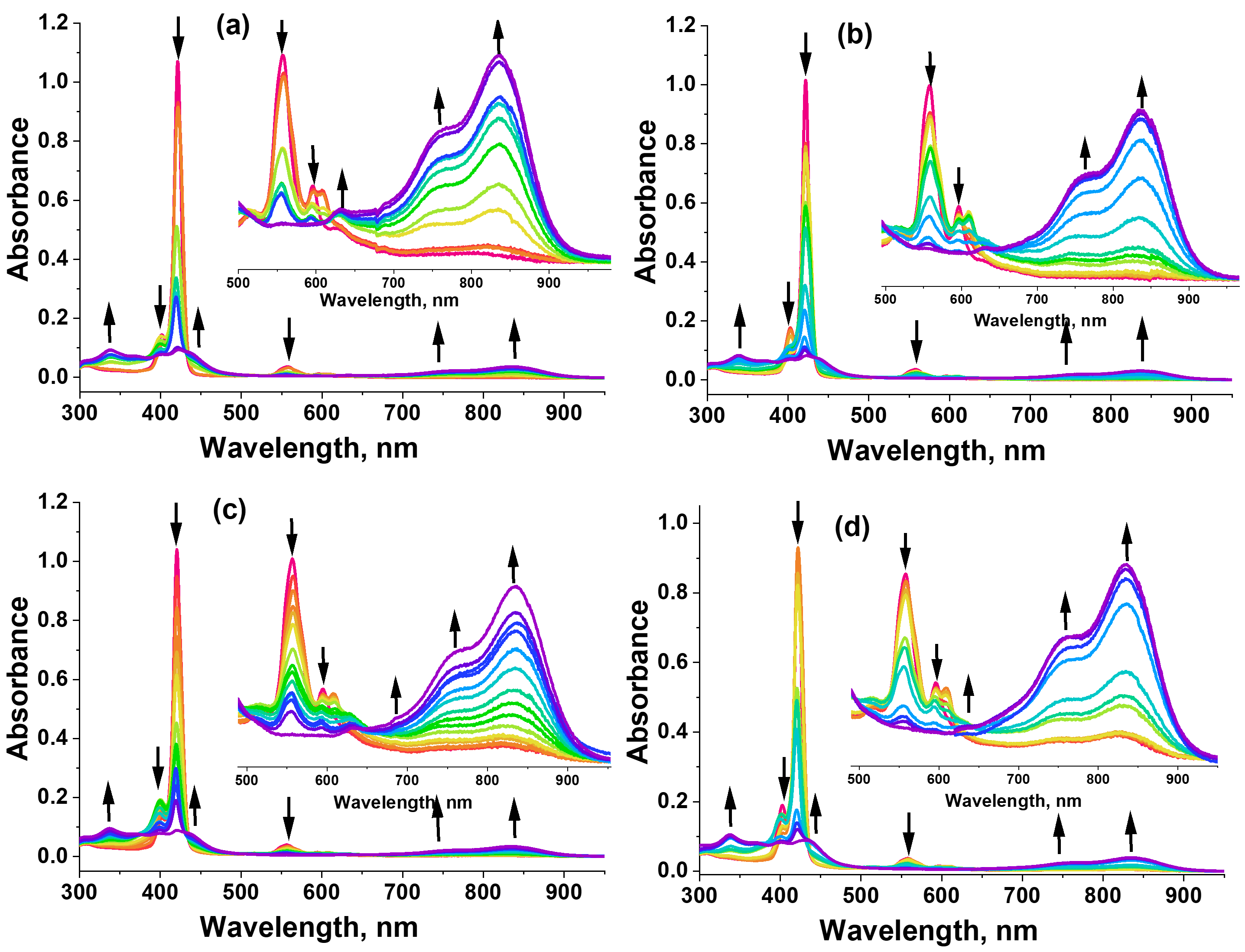

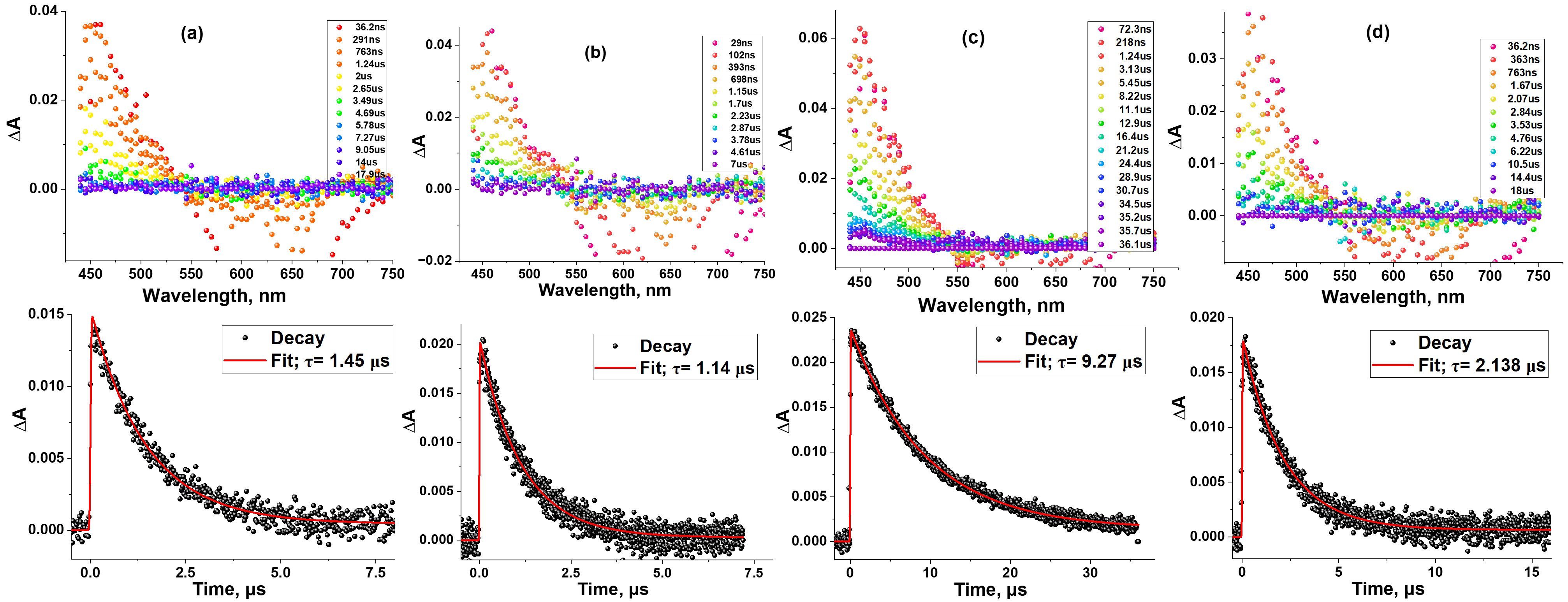
**Figure S16**. Nanosecond transient absorption spectra at the indicated delay times ([λ](https://wumbo.net/symbols/lambda/)_ex_ =415 nm) of (a) AlC3P, (b) (AlC3P)_2_, (c) AlC7P, (d) (AlC7P)_2_ in solvent *o*-DCB. Their respective triplet state decay curves (490 nm peak) with lifetime are shown in row-2.

**Figure S17**. Chemical oxidation of (a) AlC3P, (b) AlC7P, (c) (AlC3P)_2_, and (d)(AlC7P)_2_ in *o*-DCB. Chemical oxidant NOBF_4_ was used.


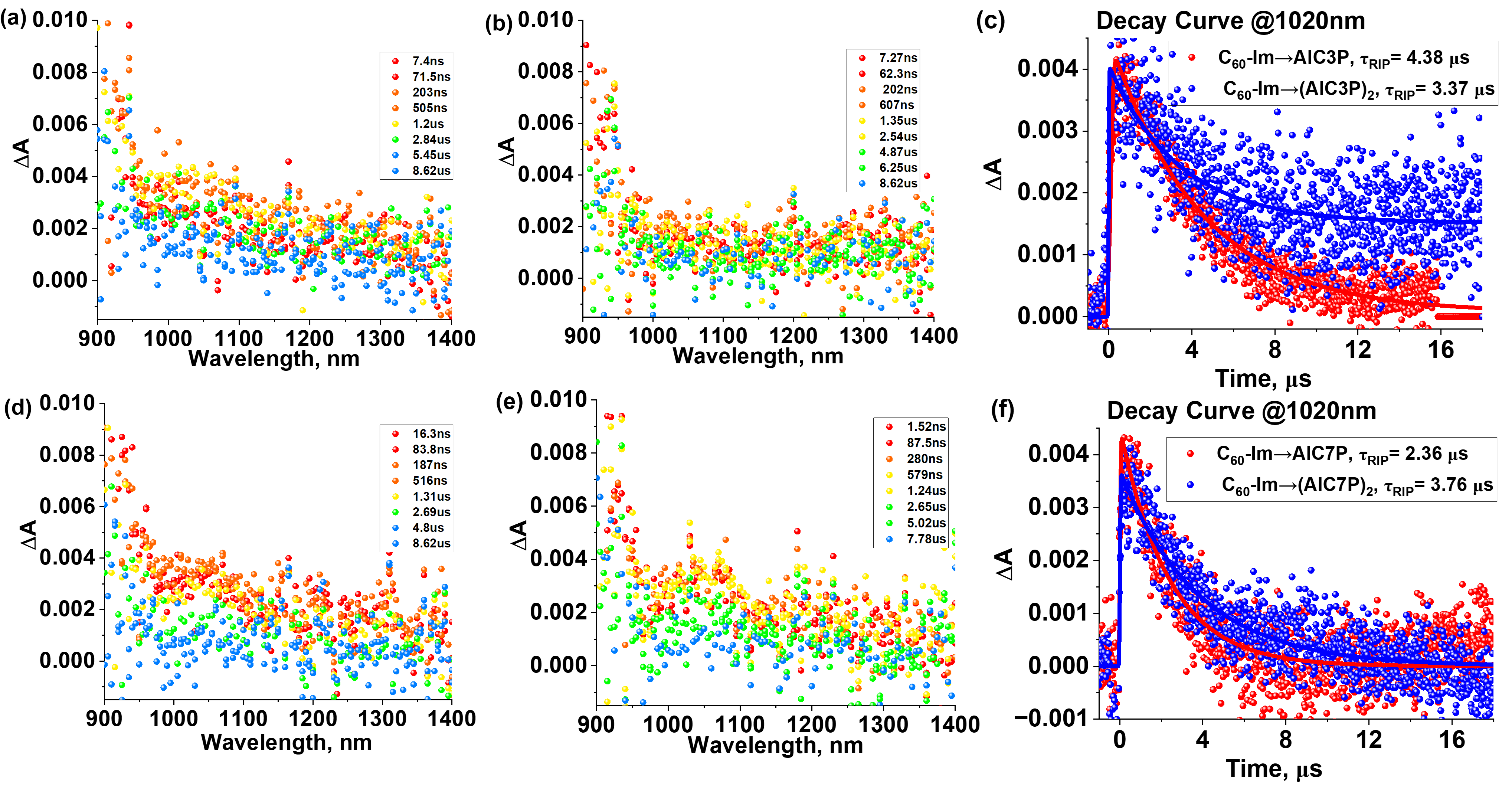


**Figure S18.** Nanosecond transient absorption spectra at the indicated delay times ([λ](https://wumbo.net/symbols/lambda/)_ex_ = 415 nm) of (a) C_60_-Im🡪AlC3P, (b) C_60_-Im🡪(AlC3P)_2_, (d) C_60_-Im🡪AlC7P, and (e) C_60_-Im🡪(AlC7P)_2_ in solvent *o*-DCB. (c) and (f) show decay profiles of 1020 nm peak (C_60_ anionic peak) with their lifetime in legends for dyads and their corresponding triads.

**Figure S19**. Spin polarized transient EPR spectra of AlC7P (black) and (AlC7P)_2_ (red) in toluene at 80 K. The dashed curves are simulations of the measured spectra. The spectra have been normalized to the same amplitude and a microwave frequency of 9.789 GHz

**Figure S20**. Spin polarized transient EPR spectra of C_60_-Im (blue) in toluene at 80 K. The dashed curve is a simulation of the measured spectrum.

**Figure S21**. Spin polarized transient EPR spectrum C_60_-Im→(AlC7P)_2_ in a narrow field range at 180 K in toluene.

**Figure S22**. Spin polarized transient EPR spectra of C_60_-Im🡪(AlC3P)_2_ at room temperature in the liquid crystal 5CB (top) and o-DCB (bottom).

**Table S2**. Parameters used to simulate the 80K TREPR spectra of C_60_-Im🡪AlC3P and C_60_-Im🡪AlC7P at 80K in Toluene shown in Figure 11.

| **Dyad** | **Component** | **Parameter** | **Value** |
| --- | --- | --- | --- |
| C_60_-Im🡪AlC3P | ^3^C_60_ | Principal g-values* | 2.0006 2.0011 2.0022 |
|  |  | ZFS parameters D(MHz), E (MHz) | –250, –23 |
|  |  | Relative zero-field level population rates (*p_x_* –*p_y_* ):(*p_y_* – *p_z_*) | –0.79 : 0.90 |
|  |  | Gaussian linewidth (mT) | 0.40 |
|  |  | HStrain (MHz) | 18, 202, 83 |
|  |  | Relative weight | 15.2 |
|  | ^3^AlPor | g-value | 2.0023 |
|  |  | ZFS parameters D(MHz), E (MHz) | 857, 213 |
|  |  | Relative zero-field level population rates (*p_x_* –*p_y_* ):(*p_y_* – *p_z_*) | –0.86 : 0.72 |
|  |  | Gaussian linewidth (mT) | 6.3 |
|  |  | HStrain (MHz) | 0 39 38 |
|  |  | Relative weight of ISC contribution | 1.65 |
|  |  | Relative weight of RP recombination contribution | 1.36 |
| C_60_-Im🡪AlC7P | ^3^C_60_ | Principal g-values* | 2.0006 2.0011 2.0022 |
|  |  | ZFS parameters D(MHz), E (MHz) | –260, –27 |
|  |  | Relative zero-field level population rates (*p_x_* –*p_y_* ):(*p_y_* – *p_z_*) | –0.70 : 0.85 |
|  |  | Gaussian linewidth (mT) | 1.0 |
|  |  | HStrain (MHz) | 0 , 161, 70 |
|  |  | Relative weight | 14.2 |
|  | ^3^AlPor | g-value | 2.0023 |
|  |  | ZFS parameters D(MHz), E (MHz) | 874, 180 |
|  |  | Relative zero-field level population rates (*p_x_* –*p_y_* ):(*p_y_* – *p_z_*) | –0.86 : 0.72 |
|  |  | Gaussian linewidth (mT) | 2.7 |
|  |  | HStrain (MHz) | 28 79 85 |
|  |  | Relative weight of ISC contribution | 2.31 |
|  |  | Relative weight of RP recombination contribution | 0.5 |
